# Genetic Transformation in Peach (*Prunus persica* L.): Challenges and Ways Forward

**DOI:** 10.3390/plants9080971

**Published:** 2020-07-31

**Authors:** Angela Ricci, Silvia Sabbadini, Humberto Prieto, Isabel MG Padilla, Chris Dardick, Zhijian Li, Ralph Scorza, Cecilia Limera, Bruno Mezzetti, Margarita Perez-Jimenez, Lorenzo Burgos, Cesar Petri

**Affiliations:** 1 Department of Agricultural, Food and Environmental Sciences, Università Politecnica delle Marche, 60131 Ancona, Italy; angela.ricci@pm.univpm.it (A.R.); s.sabbadini@staff.univpm.it (S.S.); cnlimera1983@hotmail.com (C.L.); b.mezzetti@staff.univpm.it (B.M.); 2 Laboratorio de Biotecnología, La Platina Research Station, Instituto de Investigaciones Agropecuarias, Santa Rosa, La Pintana, Santiago 11610, Chile; hprieto@inia.cl; 3 Área de Genómica y Biotecnología, Grupo de Morfogénesis y Modificación Genética, IFAPA-Centro de Churriana, Cortijo de la Cruz s/n, 29140 Málaga, Spain; isabelm.gonzalez.padilla@juntadeandalucia.es; 4 USDA-ARS-Appalachian Fruit Research Station, 2217 Wiltshire Road, Kearneysville, WV 25430, USA; chris.dardick@usda.gov (C.D.); zhijian.li@usda.gov (Z.L.); 5 Ralph Scorza LLC, Plant Breeding and Biotechnology Consulting Services, P.O. Box 1191, Shepherdstown, WV 25443, USA; ralphscorza@gmail.com; 6 Mejora Genética de Cítricos, Instituto Murciano de Investigación y Desarrollo Agroalimentario (IMIDA), C/ Mayor s/n, 30150 Murcia, Spain; mrgperezjimenez@gmail.com; 7 Departamento de Mejora Vegetal, Grupo de Biotecnología de Frutales, CEBAS-CSIC, Campus Universitario de Espinardo, 30100 Espinardo, Murcia, Spain; burgos@cebas.csic.es; 8 Departamento de Fruticultura Subtropical y Mediterránea, IHSM-UMA-CSIC, Avenida Dr. Wienberg, s/n. 29750 Algarrobo-Costa, Málaga, Spain

**Keywords:** biotechnology, organogenesis, plant breeding, *Rosaceae*, somatic embryogenesis, stone fruits

## Abstract

Almost 30 years have passed since the first publication reporting regeneration of transformed peach plants. Nevertheless, the general applicability of genetic transformation of this species has not yet been established. Many strategies have been tested in order to obtain an efficient peach transformation system. Despite the amount of time and the efforts invested, the lack of success has significantly limited the utility of peach as a model genetic system for trees, despite its relatively short generation time; small, high-quality genome; and well-studied genetic resources. Additionally, the absence of efficient genetic transformation protocols precludes the application of many biotechnological tools in peach breeding programs. In this review, we provide an overview of research on regeneration and genetic transformation in this species and summarize novel strategies and procedures aimed at producing transgenic peaches. Promising future approaches to develop a robust peach transformation system are discussed, focusing on the main bottlenecks to success including the low efficiency of *A. tumefaciens*-mediated transformation, the low level of correspondence between cells competent for transformation and those that have regenerative competence, and the high rate of chimerism in the few shoots that are produced following transformation.

## 1. Introduction

The genus *Prunus*, belonging to the family *Rosaceae*, includes a large number of fruit tree species known as “stone fruits” because the seed is encased in a hard, lignified stone-like endocarp. The edible portion of the fruit is the fleshy mesocarp, although the genus also includes nut crop species such as almond (*Prunus dulcis* Miller) where the mesocarp development is arrested. The major commercial stone fruit species are apricot (*Prunus armeniaca* L.), European plum (*Prunus domestica* L.), Japanese plum (*Prunus salicina* Lindl.), peach and nectarine (*Prunus persica* L.), sour cherry (*Prunus cerasus* L.), sweet cherry (*Prunus avium* L.), and almond.

Peach has been proposed as a model plant for the *Rosaceae* family [1] due to a relatively short juvenility period (2–3 years) compared to most of other fruit tree species, as well as its genetic characteristics including self-pollination and relatively small genome size (diploid (*n* = 8)). In the genus *Prunus*, all constructed linkage maps contain a framework of markers in common with the peach reference physical map “Texas” × “Earlygold” (T × E) [2]. Furthermore, peach was the first *Prunus* species to be sequenced. The current peach genome version (Peach v2.0) [3], generated from a doubled haploid seedling from the cultivar “Lovell”, together with the availability of new technologies for high-throughput genome and transcriptome analyses, offers new possibilities for QTL and MTL application and candidate gene identification in all *Prunus* species. Substantial progress has been made in *Prunus* genetics and genomics. The Genome Database for *Rosaceae* (GDR, https://www.rosaceae.org) provides access to all publicly available genomics, genetics, and breeding data in *Rosaceae* [4].

Almost 30 years have passed since the first published report on the regeneration of transformed peach plants [5]. Nevertheless, the general applicability of genetic transformation to this species has not yet been established. In the absence of an efficient peach transformation system, progress in determining gene function will remain slow. As an alternative, a highly efficient transformation method in European plum (*P. domestica* L.) has shown to be a useful tool for functional genomics studies in *Prunus* spp. [6]. However, peach genetic engineering is not only significant for gene function studies. The lack of efficient peach genetic transformation protocols precludes the application in peach of new biotechnological tools such as RNA interference (RNAi), trans-grafting, cisgenesis/intragenesis, or genome editing in peach breeding programs, as are currently being applied in other fruit tree species [7].

Although protocols for plant regeneration from different peach tissues (calli from immature embryos, mature and immature cotyledons, leaf explants) have been reported (e.g., [8,9,10,11]), there are only three reports on regeneration of transgenic peach plants, all from seed-derived tissues [5,12,13]. Unfortunately, none of these reports have been reproduced in other laboratories. Sabbadini et al. [14] reported the regeneration of two transgenic lines from somatic tissues of the *P. persica x Prunus amygdalus* hybrid “GF677”. More recently, Xu et al. (2020) published an *A. rhizogenes*-mediated transformation method for peach hypocotyl, leaf, and shoot explants to generate transgenic hairy roots to produce composite plants with wild-type shoots and transgenic roots.

Many strategies have been tested in order to obtain an efficient peach transformation system. Despite the amount of time and the efforts invested, the lack of success has meant that much data, potentially useful to the scientific community, has not been published. This review is the result of a collaboration of scientists from different laboratories throughout the world. Here, we present an overview of peach regeneration and transformation research and describe novel strategies and procedures undertaken at our facilities aimed at producing transgenic peaches. Possible future studies and approaches are discussed.

## 2. State of the Art Work in Peach Transformation

The development of a system for gene transfer or gene editing in peach depends upon the availability of effective regeneration procedures coupled with techniques that permit efficient DNA delivery, selection of transformed tissues, and recovery of transgenic plants. Unfortunately, *P. persica* is universally known to be one of the most recalcitrant species in terms of production of transformed plants [13]. Table 1 summarizes the results published to date on peach genetic transformation. To the best of our knowledge, other than these published results, Okanagan Specialty Fruits (OSF) Inc. (Summerland, BC, Canada) achieved success in the 2000s through developing some peach transformed lines with a procedure that involved somatic embryogenesis (SE). However, the efficiency of the technique was very low, and their success was based on the extremely high number of explants used. Currently, the company has abandoned this line of research (John Armstrong, personal communication).

### 2.1. Type of Explant

There are two classes of explants that may be used for regeneration of transformed plants: juvenile material (seed-derived tissues) or adult material. Regeneration from adult somatic tissues is highly recommended for clonally propagated crops in order to maintain genetic uniformity of the cloned plants, especially for the highly heterozygotic *Prunus* species. A procedure that allows the genetic transformation of a range of clonally propagated genotypes would be the ideal situation, not only for peach but for any woody fruit species. Unfortunately, procedures that use clonal tissues as the source of explants cannot be readily transferred among genotypes. Several reports [11,21,23,24,25] showed the difficulty in establishing a standard protocol for peach leaf organogenesis. Typically, these protocols are highly genotype-dependent and are influenced by the combination of factors such as the type and age of starting donor explant, basal medium composition, dark/light period during culture, and plant growth regulators supplemented to basal culture medium. Despite these above-mentioned regeneration studies, there are no routine genetic transformation systems reported for any peach genotype.

Currently, most of the transformation procedures in *Prunus* spp. involve the use of seed-derived explants, including apricot [26,27], European plum [28,29,30], and Japanese plum [31]. While transformation of peach from seed explants has been reported [5,12,13] the successes have not been repeated in other laboratories. If a routine transformation method for peach seed-derived tissues were to be developed, it could have an impact on the development of new rootstock varieties, and it would also allow for the introduction of novel genes into the peach germplasm that could be used in conventional breeding programs, especially in view of the relatively short generation time for peach.

### 2.2. DNA Delivery Method

Ye et al. [17] optimized biolistic parameters for peach. Bombardment was applied to different tissues, but transformation was stable only in the zygotic embryo-derived calli. They obtained 65 putative transformed calli lines, 19 of these produced shoot-like structures, but shoots were not recovered.

Several studies have investigated factors affecting *Agrobacterium*-mediated gene transfer in peach. Different peach tissues, such as embryogenic calli, leaves, and immature embryos, are amenable to *A. tumefaciens*-mediated transformation [15]. *Agrobacterium*-mediated transformation of multiple types of explants from different genotypes using diverse bacteria strains harboring different plasmids have been evaluated [18]. The combinations utilized had a strong influence in the percentage of infected explants expressing the reporter genes green fluorescent protein (*gfp*) or β-glucuronidase (*gus*), suggesting that it will be necessary to adjust strain/plasmid/promoter/vector with each type of explant to optimize transformation and regeneration efficiencies. In that study, seed-derived internodes showed the highest transformation percentages of 56.8% and 26.0% on the basis of GUS or GFP detection, respectively, compared to other explants such as cotyledons, leaves, or embryonic axes [18]. Zong et al. [21] found the strain EHA105 as the most efficient for transient transformation in the peach–almond hybrid rootstock “Hansen 536” leaves, compared to GV3101 and LBA4404.

Zimmerman and Scorza [32] reported on the success of a procedure combining biolistic and *A. tumefaciens* for the transformation of tobacco meristems and the production of transgenic plants. However, when tested on peach, they encountered a significant mortality rate due to the mechanical damage and desiccation during dissection to expose the meristems. In addition, bacterial growth was difficult to control [33].

### 2.3. Transgenic Peach Plant Recovery

As stated previously, currently there are only three publications reporting the regeneration of transgenic *P. persica* plants [5,12,13]. The first report utilized a “shooty mutant” strain of *A. tumefaciens*. This strain carried a Ti plasmid with a functional isopentenyl phosphotransferase gene *(ipt)*, involved in cytokinin biosynthesis, and a Tn5 transposon-inactivated auxin biosynthesis gene (*iaaM*). The infection with a “shooty mutant” strain induces the development of tumors, from which transgenic shoots regenerate. Peach tissues transformed with the *ipt* gene allowed selection of transformed shoots on a medium low in plant growth regulators (PGRs). In vitro assays of these plants demonstrated delayed senescence on cytokinin-free medium as compared with non-transformed controls. The resulting peach plants were shorter in stature than controls, and one line exhibited greater branching, presumably due to the effect of the *ipt* transgene expression [34].

Pérez-Clemente et al. [12], using longitudinal mature embryo slices as the explant source, reported the regeneration of transgenic plants expressing the *npt*II, which confers resistance to aminoglycoside antibiotics and *gfp* marker genes with a transformation efficiency of 3.6 ± 1.0%. This protocol improved upon the preceding report of Smigocki and Hammerschlag [5] in that mature embryos are available year-round while immature embryos are available for only a limited time each year.

The most recent report relies on a procedure using SE from immature peach cotyledons. It describes the production of transformed plants expressing *gfp* from “O’Henry” and “Rich Lady” immature cotyledons with a transformation efficiency of about 0.6% [13].

Sabbadini et al. [14] reported an *A. tumefaciens*-mediated transformation protocol using “GF677” (*P. persica x P. amygdalus*) meristematic bulk (MB) slices as starting material. Meristematic bulks (MBs), initiated from shoot tips, mechanically and chemically treated, differentiated, and regenerated adventitious shoots. After 32 weeks of selection with kanamycin, the efficiency of the procedure was relatively poor (0.3%), and when this methodology was applied to “Hansen 536”, another peach x almond hybrid rootstock, only produced transgenic callus lines [20].

### 2.4. Methodologies for Functional Genomics Studies

Honda and Moriguchi [19] described a protocol for transient gene expression analysis using protoplasts isolated from immature peach fruits. Xu et al. [22] developed an *A. rhizogenes*-mediated transformation method to generate transgenic hairy roots from peach shoots to produce composite peach plants with transgenic roots and non-transgenic shoots. They proposed this method for studying root–rhizosphere microorganism interactions in peach and as a method for clonal propagation. The authors also demonstrated the applicability of the system to assess endogenous gene functions. Regarding methodologies for functional genomics studies in peach, it is interesting to note the induction of RNA silencing through a *Prunus* necrotic ringspot virus (PNRSV) viral vector for virus-induced gene silencing (VIGS) [35]. They demonstrated that the PNRSV-based vector could efficiently silence endogenous genes in peach.

## 3. Further Approaches Applied to Improve Peach Regeneration-Transformation

This section summarizes the results of different strategies/procedures aimed at producing transgenic peaches performed at our facilities (Table 2). Although we are working in laboratories throughout the world, we have collaborated in the past and continue active cooperation in order to obtain an efficient peach transformation system. Our approaches have thus far failed at developing a robust peach transformation protocol. Nevertheless, our findings represent incremental progress towards this goal and are potentially useful to the scientific community. In the subsequent discussion, the work of this group is presented without identifying individual collaborators.

### 3.1. Assessed Methodologies that Involve Peach Juvenile Tissues

#### 3.1.1. SE from Juvenile Tissues

The culture of seed-derived explants can be considered as a first stage in the protocol leading to SE. Seed maturity stage and genotype affect the induction and/or the development of the organized structures [36,37,38]. SE has been initiated from friable callus [8], longitudinal cotyledonary slices [39], and cultured immature zygotic embryos [38]. We have observed that immature cotyledons (50 to 70 days post-bloom) cultured in LP medium [11] and supplemented with 6-benzylaminopurine (BAP; 5.0 µM) and α-naphthaleneacetic acid (NAA; 3.0 to 5.0 µM) led to consistent SE production in “O’Henry”, “Elegant Lady”, “Rich Lady”, and “Venus” peaches. Although genetic transformation was not evaluated, this procedure (Figure S1) allowed whole-plant production in the above-mentioned genotypes.

#### 3.1.2. Organogenesis from Juvenile Tissues

##### Immature Cotyledons

**Figure 1:**
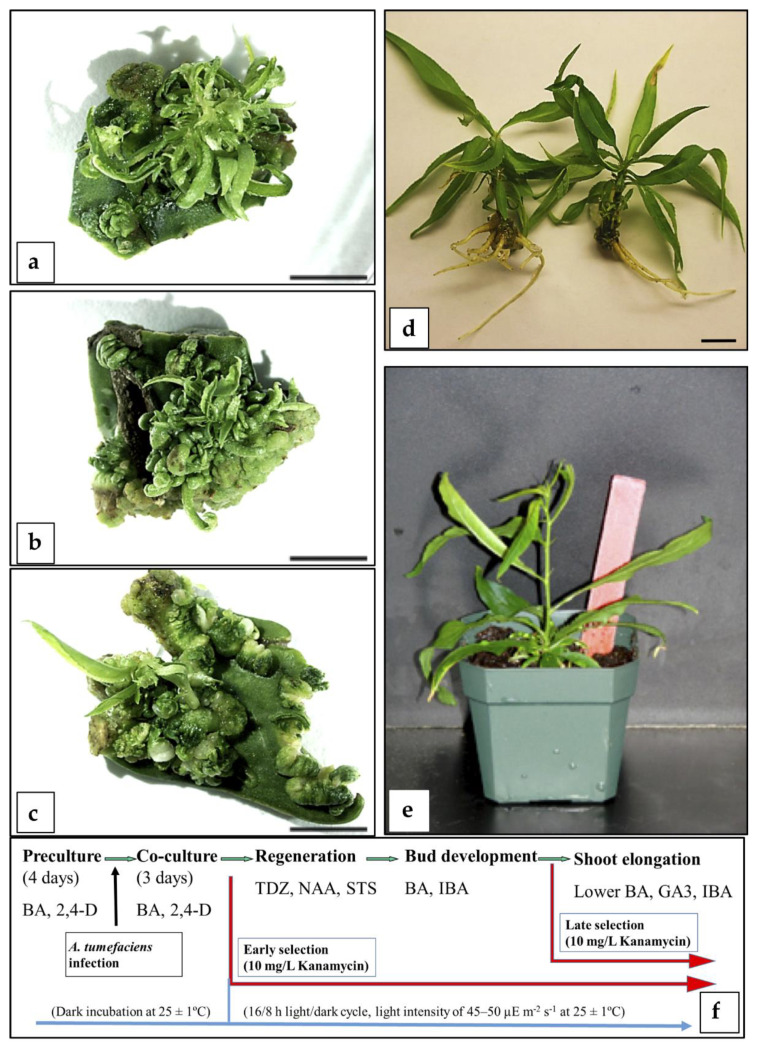
Direct adventitious regeneration from immature peach cotyledons: (**a**–**c**) Bud regeneration observed in immature cotyledons of “Starlite”, “Bailey”, and “Guardian”, respectively, under no selection regime and controls after 5–6 weeks from the beginning of the experiment (bar = 0.5 cm). (**d**) Rooted shoots after 4 weeks in rooting medium prepared for acclimatization (bar = 1 cm). (**e**) Potted plant cultured in a greenhouse after the rooting and acclimatization process. (**f**) Scheme of the methodology followed for regeneration of transformed shoots.

A regeneration protocol for “Bailey”, “Guardian”, and “Starlite” immature cotyledons produced around 80% regeneration (Figure 1 and Procedure S1). The addition of 60 µM silver thiosulphate (STS) to the regeneration media and a two-step strategy to recover the buds allowed the successful regeneration, development, elongation, and further establishment of adventitious shoots in a greenhouse (Figure 1), significantly improving the results compared to those previously reported [38].

This regeneration protocol (Procedure S1) was combined with *Agrobacterium*-mediated transformation and two different selection strategies (Figure 1f): an early selection, applying 10 mg/L kanamycin right after the co-cultivation, and a late selection, where kanamycin (10 mg/L) was applied at the elongation stage. Immature cotyledons of “Starlite”, “Bailey”, and “Guardian” were infected with *A. tumefaciens* GV3101 harboring the pVNFbin binary plasmid [40]. On the basis of PCR analysis with specific primers for the *gus* gene, a total of 21 putative transgenic shoots were obtained. Only a few clones survived the entire selection procedure and were transferred to a greenhouse. Subsequent molecular tests (Southern blot analysis) showed that all were escapes (not shown). These results suggested that neither selection strategy allowed the survival of non-transformed or chimerical plants. In future studies, the application of a gradual increasing selection strategy might eliminate the recovery of chimeric plants and non-transformed escapes.

##### Mature Seed Hypocotyl Slices

Different factors affecting adventitious regeneration from hypocotyl slices were studied such as basal media; gelling agents; different types, concentrations, and combinations of PGRs; 2,4-dichlorophenoxyacetic acid (2,4-D) pulses; dark induction periods; addition of ethylene inhibitors, such as STS or 2-aminoethoxyvinyl glycine (AVG); polyamines; and coconut water.

**Figure 2:**
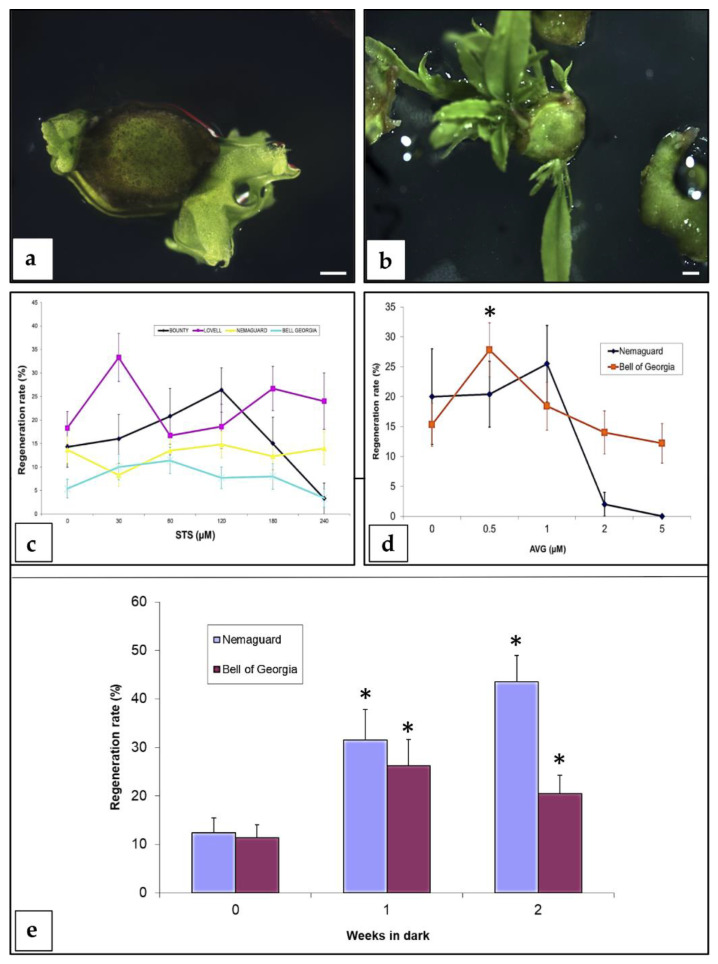
Factors affecting adventitious regeneration from peach mature seed hypocotyl slices. (**a**) First buds appearing after 4 weeks of culture from the beginning of the experiment (bar = 1 mm). (**b**) Adventitious regeneration from a peach hypocotyl section after 6 weeks of culture from the beginning of the experiment (bar = 1 mm). (**c**) Effect of silver thiosulphate (STS) on regeneration. A total of 182, 498, 862, and 700 explants were used in this experiment for “Bounty”, “Lovell”, “Nemaguard”, and “Bell of Georgia”, respectively. Vertical bars indicate standard errors (SE). (**d**) Effect of 2-aminoethoxyvinyl glycine (AVG) on regeneration. A total of 229 and 484 explants were used in this study for “Nemaguard” and “Bell of Georgia”, respectively. Vertical bars indicate SE. Asterisks indicate significant regeneration increased (*p* < 0.01) compared to the control without addition of AVG, according to Pearson’s chi-test. (**e**) Effect of dark incubation period on regeneration. A total of 255 and 326 explants were used in this study for “Nemaguard” and “Bell of Georgia”, respectively. Vertical bars indicate SE. Asterisks indicate statistical significance (*p* < 0.01) compared to the treatment without dark induction, according to Pearson’s chi-test. All the experiments were repeated at least twice.

Adventitious buds were observed as direct organogenesis after 4 weeks of culture from the beginning of the experiment, and additional buds appeared in the subsequent 2–3 weeks (Figure 2a,b). Most of the factors studied did not increase or affect adventitious regeneration. Results showed that QL basal salts [41] slightly increased regeneration rates compared to MS salts [42] (data not shown). The effect of the ethylene inhibitors (STS and AVG) was genotype-dependent (Figure 2c,d). A synergistic effect was not found when both ethylene inhibitors were added to the regeneration medium (data not shown). A dark induction period appeared to be important in peach organogenesis from mature hypocotyl explants. One or two weeks in the dark significantly increased (*p* < 0.01) the regeneration rate for both of the cultivars tested, “Nemaguard” and “Bell of Georgia” (Figure 2e). Because of this study, the most appropriate conditions for adventitious regeneration from peach mature seed hypocotyl explants were determined (Procedure S2). Regeneration rates were 32% for “Bailey”, 28% for “Bell of Georgia”, 34% for “Bounty”, 33% for “Lovell”, 43% for “Nemaguard”, and 14% for “TruGold”.

**Figure 3:**
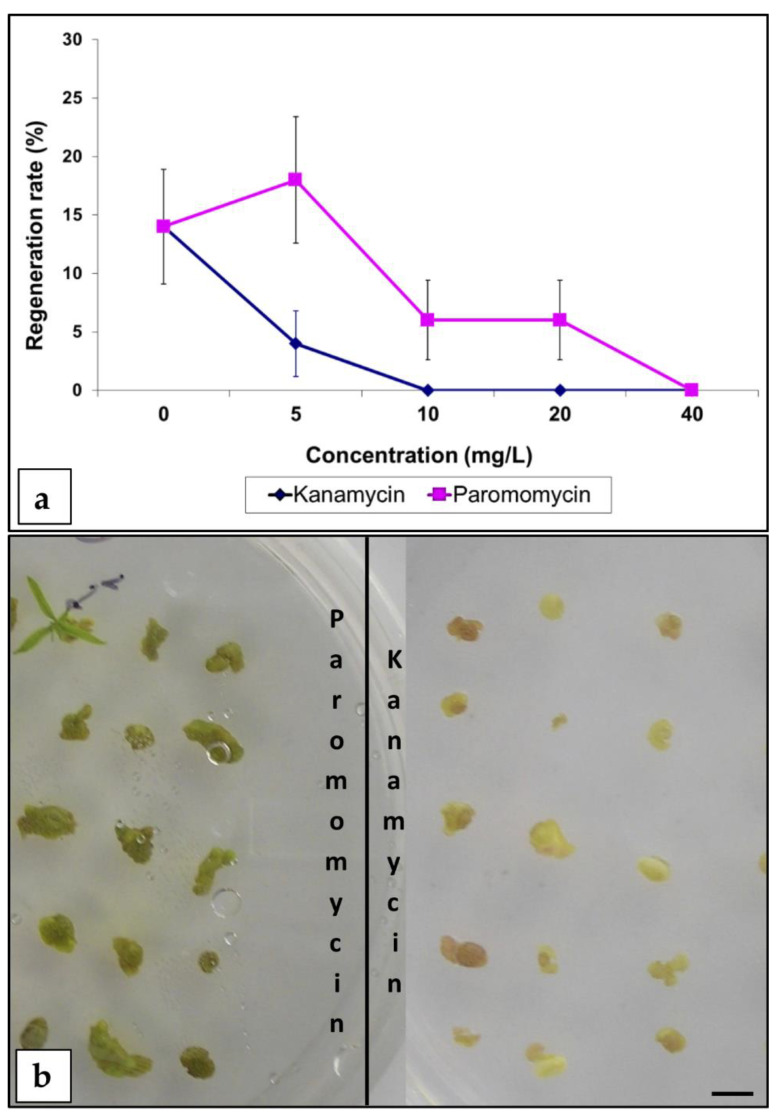
Effect of aminoglycoside antibiotics (paromomycin and kanamycin) on mature peach seed hypocotyl sections. (**a**) Effect on adventitious bud regeneration. For this study, 450 explants were used (cv. “Bell of Georgia”). The experiment was repeated at least twice. Bars indicate SE. (**b**) Explants incubated in regeneration medium containing 20 mg/L of the specified antibiotic after 5 weeks of culture (bar = 0.5 cm).

**Figure 4:**
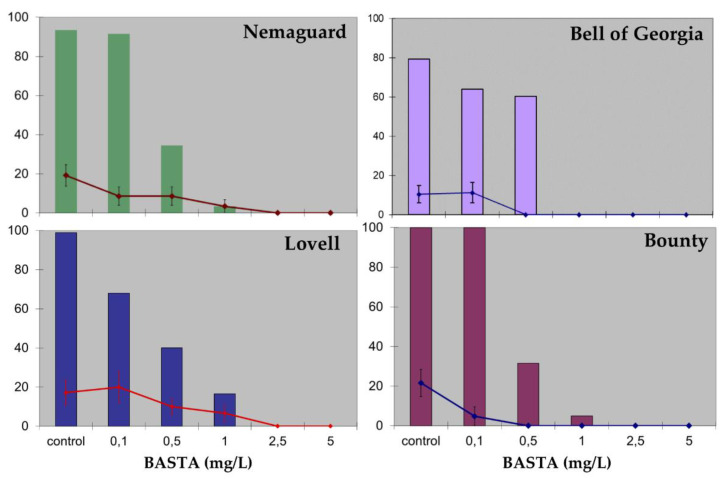
Effect of BASTA herbicide on mature peach seed hypocotyl sections. Color column charts represent explant survival (%) after 2 weeks from the beginning of the experiment. Line charts represent regeneration rates (%) after 7 weeks from the beginning of the experiment with the vertical bars indicating SE. A total of 177, 264, 132, and 183 explants were used in this experiment for “Nemaguard”, “Bell of Georgia”, “Lovell”, and “Bounty”, respectively. Experiments were repeated at least twice.

Following this regeneration method (Procedure S2), two different selection strategies were considered: (i) an aminoglycoside antibiotic-based selection strategy, or (ii) selection with the herbicide BASTA. Experiments to establish the inhibitory concentrations of the selective agents for the different peach cultivars were performed, and regeneration inhibition curves for aminoglycoside antibiotics (kanamycin and paromomycin) and BASTA herbicide were established (Figure 3 and Figure 4). A total of 10 mg/L of kanamycin or 40 mg/L of paromomycin were necessary to inhibit regeneration (Figure 3a). When paromomycin was added to the media, explants looked healthier than in the presence of kanamycin (Figure 3b). Peach hypocotyl sections appeared very sensitive to the herbicide BASTA. Regeneration inhibitory concentrations varied among the genotypes tested, being 0.5 mg/L for “Bell of Georgia” and “Bounty”, 1.0 mg/L for “Nemaguard”, and 2.5 mg/L for “Lovell” (Figure 4). The two highest concentrations tested (2.5 and 5.0 mg/L) were highly toxic, and all explants exposed to them died (Figure 4).

In other woody species, such as apple and apricot, it has been suggested that substantial necrosis in non-transformed tissues under selection pressure could inhibit regeneration from transformed cells [43,44]. As illustrated in Figure 3b, non-transformed hypocotyl sections cultured in the presence of paromomycin remained green, suggesting that this could be a more appropriate selective antibiotic than kanamycin for this peach explant. Following a similar strategy, 0.1–0.5 mg/L of BASTA was established as the proper selective concentration (depending on the genotype), severely reducing regeneration but allowing explant survival.

A set of experiments was carried out, with the *A. tumefaciens* strains EHA101 and GV3101 harboring the pVNFbin binary plasmid [40] containing the selective marker gene *npt*II. Another set of experiments was performed with the *A. tumefaciens* strain EHA105 harboring the pBarGUS plasmid [45], the *bar* selective marker gene conferring resistance to BASTA herbicide. Both plasmids contained an intron-containing *gus* gene, which prevents expression of the gene by bacteria, as the transformation reporter gene. After 3 days of co-cultivation, infected hypocotyl explants (cv. “Bailey” and “TruGold”) were placed in selective medium containing 40 mg/L paromomycin (*npt*II-transformed tissues) or 0.1 mg/L BASTA (cv. “Nemaguard”) (*bar*-transformed tissues). Stable transformation was evaluated with histochemical *gus* assays [46] after 7 weeks from the beginning of the experiments. There were low transformation rates for both constructs used. On average, 20% of the infected explants showed only a few GUS spots on their surface. Regeneration rates in the infected explants were similar to those observed in the non-transformed explants (Figure 3a and Figure 4), and transgenic “blue” shoot buds were not observed. This work indicated that negative selection strategy was not appropriate.

In addition, a positive selection strategy was tested. In this case, selection was not based on toxicity for non-transformed tissues. Using a positive selection strategy, transformed cells have an advantage over non-transformed cells, allowing them to proliferate and differentiate into new adventitious buds. Following an approach similar to Smigocki and Hammerschlag [5], *ipt* was used as the selective marker gene. Hence, only *ipt*-transformed cells should be able to regenerate in a cytokinin-free or low-level regeneration medium.

Following the regeneration procedure described above (Procedure S2), we compared the effect of the thidiazuron (TDZ) concentration on the organogenesis of “Bailey” hypocotyl sections among non-infected and infected explants using the *A. tumefaciens* EHA105 strain harboring the *ipt*-containing construct. The effect of the *ipt* gene was evident on adventitious root regeneration (Table 3). The frequency of root regeneration was clearly reduced on infected explants compared to the non-infected controls (Table 3) indicating that the IPT enzyme increased the cytokinin to auxin ratio. However, a marked effect of *ipt* on shoot regeneration relative to controls was not observed for any of the TDZ concentrations tested (Table 3). A total of 65 putative transgenic buds were isolated and elongated. Molecular analyses (PCR and/or DNA blot) revealed that all of them were escapes.

##### Seed-Derived Internodes

**Figure 5:**
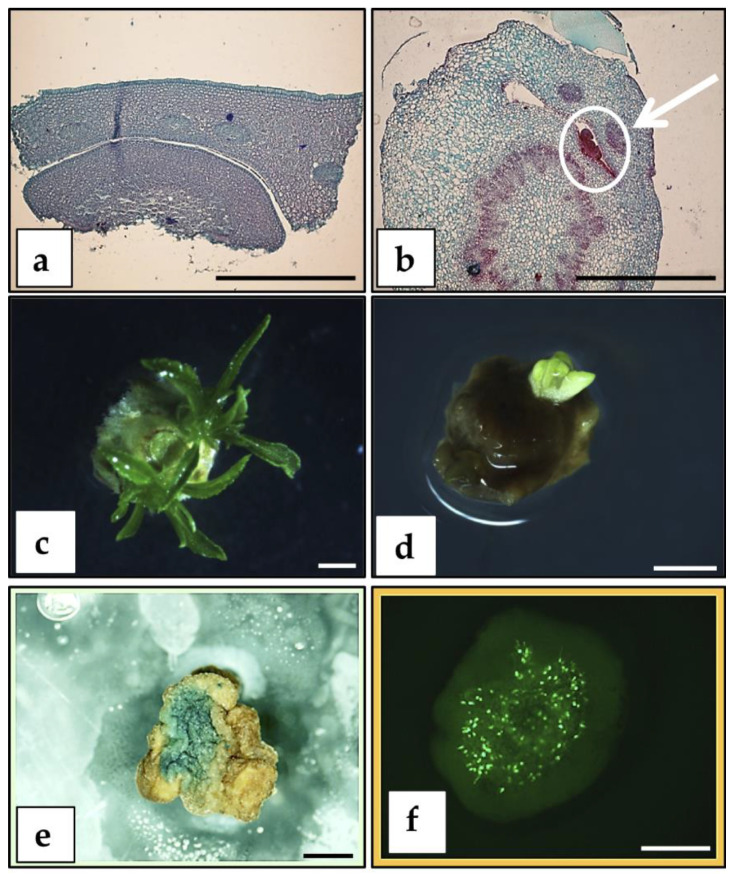
Regeneration and transformation from peach seed-derived internode explants. (**a**) Histological study of the internode/cotyledon attachment area at day 0 (bar = 1 mm). (**b**) Histological study of the internode/cotyledon attachment area at day 9. Internode with axillary bud with evident meristematic dome with leaflets growing from the epidermis (arrow) (bar = 1 mm). (**c**) Adventitious shoot regeneration from a peach internode explant (bar = 1 mm). (**d**) Adventitious shoot regeneration from a EHA101 pVNFbin-infected explant cultured in regeneration medium supplemented with 20 mg/L paromomycin (bar = 1 mm). (**e**) β-Glucuronidase (GUS) activity in a peach internode explant (bar = 1 mm). (**f**) Green fluorescent protein (GFP) activity in a peach internode explant (bar = 1 mm).

Following the study of Padilla et al. [18], where seed-derived internodes showed the highest *Agrobacterium*-mediated transformation rate, we examined the organogenic potential of these explants. Histological studies demonstrated the absence of preformed buds or shoot primordia in the explants at day 0 (Figure 5a), whereas at day 9, meristematic domes with leaflets were observed as emerging from the epidermis of the internode (Figure 5b). The best regeneration rates (42.9%) for “Bailey” explants were reached when 10.0 µM BAP was added to the medium and seeds were germinated in the presence of 40.0 µM BAP (Table 4). Shoots regenerated from the central part of the explant (Figure 5c), coinciding with the area of greater GUS and GFP expression (Figure 5e,f). Following this regeneration protocol (Procedure S3), we carried out *Agrobacterium*-mediated transformation experiments with the EHA101 disarmed strain containing the pVNFbin binary plasmid. Selection with 20 mg/L paromomycin was applied right after a 2-day co-cultivation. Paromomycin at 20 mg/L inhibited regeneration from non-infected explants. On the other hand, 12.2% of the infected explants showed shoot regeneration (Figure 5d) and around 30% of the assayed explants showed few GUS spots. Transgenic shoots were not obtained since all regenerated buds died during selection.

### 3.2. Assessed Methodologies that Involve Peach Adult Tissues (Cultivars or Rootstocks)

#### 3.2.1. SE from Peach Adult Clonal Material

To the best of our knowledge, somatic embryos from peach mature tissues have not been obtained to date. The aim of the study described here was to obtain a protocol for SE from different peach mature tissues.

##### SE from Leaf Explants

**Figure 6:**
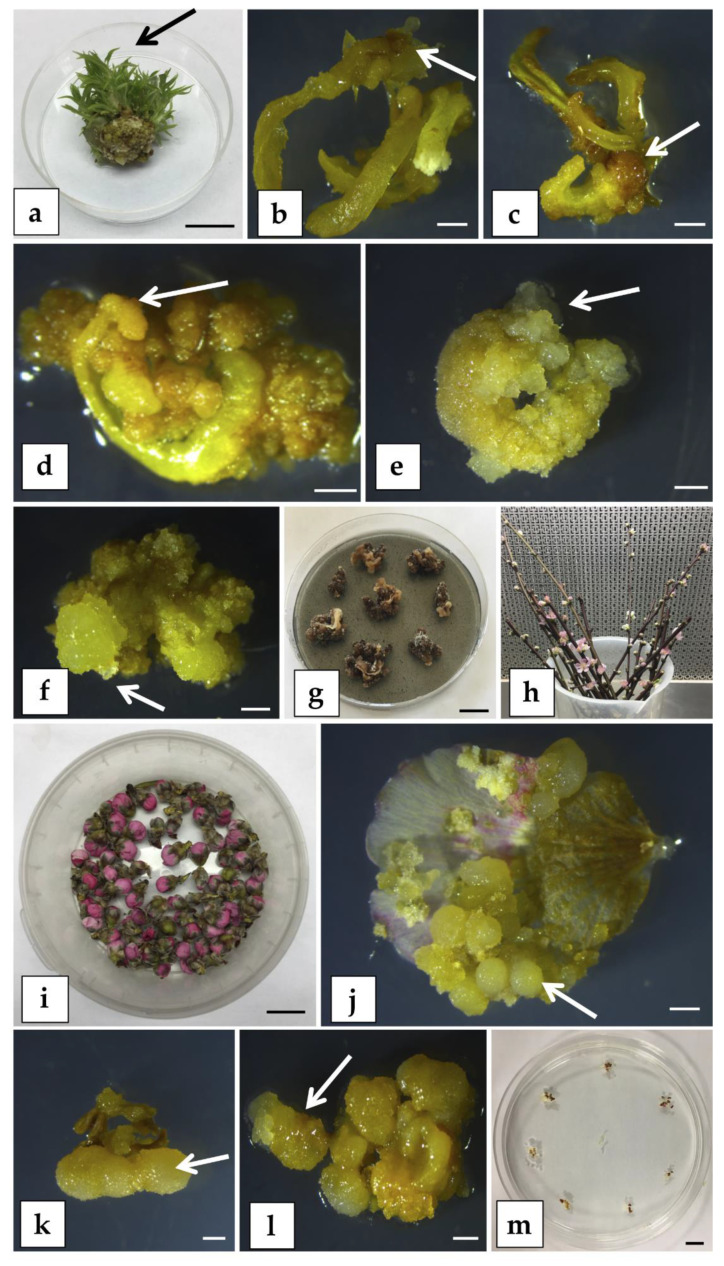
Somatic embryogenesis (SE) trials on peach mature explants. (**a**) In vitro meristematic bulk (MB) of “Hansen 536”; the arrow indicates the type of young leaf collected and used as starting explant in SE induction experiment (bar = 1 cm). Brownish calli (arrow) developed from “Hansen 536” leaves cultured on medium C (**b**) and on medium D (**c**); the images were taken after 3 months from the beginning of the experiment (bar = 2 mm). Cream-colored calli (arrow) developed from “Hansen 536” leaves cultured on medium A (**d**), on medium B (**e**), and on medium E (**f**); the images were taken after 3 months from the beginning of the experiment (bar = 2 mm). Necrotic calli from “Hansen 536” leaves cultured on plant growth regulator (PGR)-free medium (**g**) after 4 months from the beginning of the experiment (bar = 1 cm). Cuttings of peach rootstock “GF677” (**h**) and sterile unopened flowers of “GF677” (**i**) used as starting explants in the SE induction experiment (bar = 1 cm). Cream-colored calli (arrow) developing from petal (**j**) and anther with filament (**k**) of “GF677”, both cultured on PAM medium after approximately 3 months from the beginning of the experiment (bar = 2 mm). (**l**) Cream-colored calli formation (arrow) from “GF677” anther with filament cultured on PIV medium [47] (bar = 2 mm). (**m**) “GF677” anthers with attached filament cultured on MSI medium [52] for 3 months (bar = 1 cm).

Young leaves from in vitro meristematic bulks (MBs) of the commercial peach rootstock “Hansen 536” *(P. persica x P. amygdalus)*, obtained following the protocol described by Sabbadini et al. [20], were used as starting explants (Figure 6a). Leaves were cultured with the abaxial side in contact with the SE induction media (Procedure S4), supplemented with several combinations of PGRs and amino acids (Table SM1). In these experiments, there was no SE induction from “Hansen 536” on any of the media tested, even though differences in the frequency of caulogenesis were observed. When “Hansen 536” leaves were cultured on media C and D (Table S1), a low percentage of explants (about 15%) produced brownish calli (Figure 6b,c) after 10–12 weeks of culture. Explants placed on media A, B, and E (Table S1) produced a high percentage (about 90%) of cream-colored calli after 10–12 weeks of culture (Figure 6d–f). The cream-colored calli were transferred to a PGR-free medium (Procedure S4) in anticipation of the development of proembryonic masses and eventually somatic embryos but the calli turned brown and became necrotic 4 weeks after transferring to the PGR-free medium (Figure 6g).

##### SE from Petals and Anthers

One-year-old dormant cuttings of the peach rootstock “GF677” (*P. persica x P. amygdalus)* were used for induction of SE from unopened flowers petals and anthers (Figure 6h,i). When petals and anthers with filaments were cultured on PAM (Procedure S5), both explants produced a significant percentage (about 71% and 96%, respectively) of cream-colored calli after 10–12 weeks of culture (Figure 6j,k). The cream-colored calli were transferred onto PGR-free medium and turned brown and became necrotic after 4-6 additional weeks. When anthers with attached filaments were cultured on PIV medium [47], cream-colored calli formation was about 97% after approximately 3 months of culture (Figure 6l). However, as observed in the previous trials, they turned brown and became necrotic after 6 weeks of culture in PGR-free medium. There was no calli formation from anthers and filaments on MSI medium [48] and the explants shriveled and dried up (Figure 6m).

Our results showed that none of the media tested induced the embryogenic potential in the somatic cells treated. Studies on the evaluation of endogenous hormonal levels of peach adult tissues such as leaves and flowers would be helpful in assessing the appropriate synthetic hormonal stimulus capable of inducing SE in peach in vitro cultures. Concerning the choice of the source of explant used to obtain peach somatic embryos, various aspects must be considered. In general, the age of tissue has an impact on SE in horticultural plants [49]. The endogenous hormonal balance of *P. persica* cotyledons influenced their capacity to pass through from the differentiated to the embryogenic stage [50]. Ji et al. [49] remarked that a young tissue at early stages of development, with a high level of basal metabolism, seems to be more susceptible for SE induction compared to an older differentiated tissue. In fact, SE induction can occur only if a differentiated cell regains its totipotency [51]. As reviewed by Druart [37], SE induction in immature tissues occurs over a very short period during bloom and seed or embryo development. Thus, it would be worth trying to work on the stimulation of SE in adult tissues cultured in vitro (such as leaves, for instance), which apparently should not need to be treated in a strictly fixed period. Culture of petals and anthers at different developmental stages should be evaluated for SE production in peach, as has been carried out in species such as *Vitis* [48].

#### 3.2.2. Organogenesis from Adult Material

##### Leaf Explants

We obtained high levels of in vitro adventitious root regeneration from leaves (Table 5). This contrasts with the difficulty of regenerating shoots from peach leaf explants. Adventitious rooting was produced from leaves excised from greenhouse or in vitro-grown peach plants on MS medium [42] with 9–12 µM NAA, with or without kinetin at 0.4–1.2 µM (Procedure S6). Higher numbers of roots were obtained when leaf explants were cultured in the dark. Kinetin levels of 3.6 and 10.8 µM inhibited rooting. Roots produced in the light were thick, long, and geotropic, while roots developed in the dark were thin and non-geotropic. Roots originated from vascular areas of the leaf pieces. Root meristems were evident within 14 days after culturing leaves from plants grown in the greenhouse.

##### Efficient Shoot Proliferation and Axillary Meristematic Explants

The utilization of axillary shoot meristematic tissues as gene delivery targets may facilitate the development of a reproducible and reliable transformation system in peach. One of the major challenges of this approach is the slow rate of cell growth and shoot proliferation and the limited availability of proliferative or meristematic tissues for *Agrobacterium* infection.

**Figure 7:**
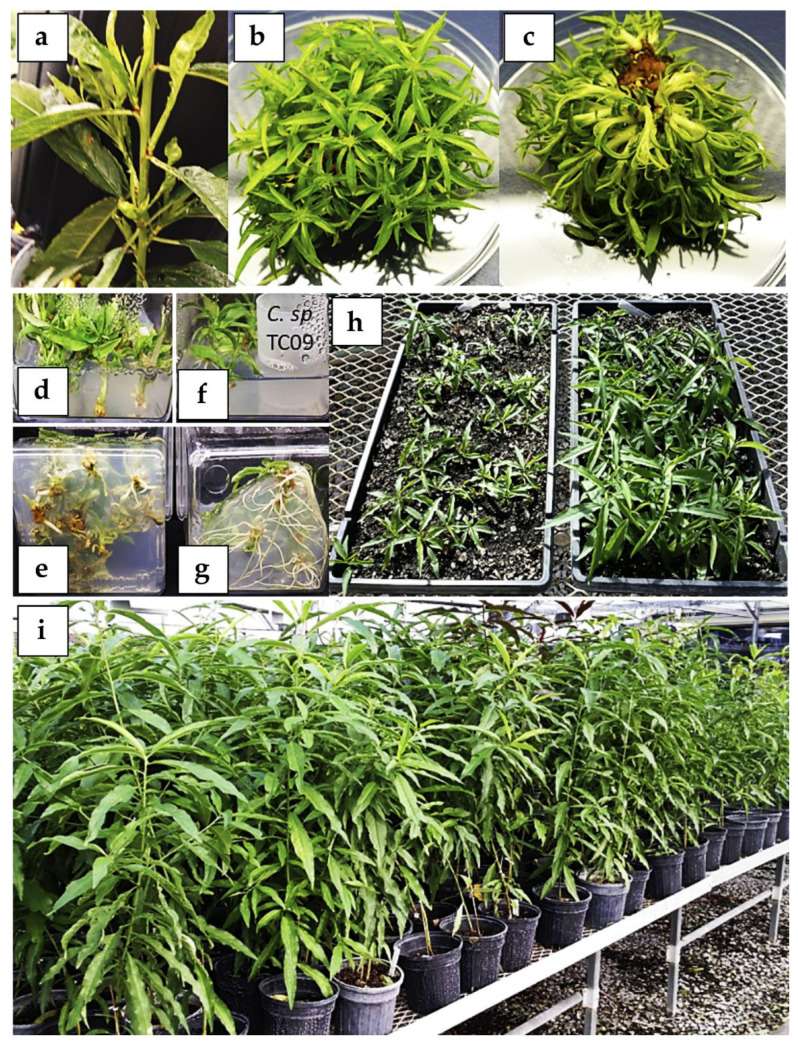
In vitro micropropagation of peach (*P. persica*) rootstock cv. “Bailey-OP”. (**a**) Shoot culture explant source from greenhouse-grown plant. Top (**b**) and bottom (**c**) views of an individual in vitro shoot cluster of peach rootstock “Bailey-OP” derived from a single shoot explant after 50 days of cultivation on LP medium supplemented with 4.5 µM 6-benzylaminopurine (BAP) and 0.5 µM IBA. Rooted shoot without (**d**,**e**) or with (**f**,**g**) exposure to VCs emitted by *C. sphaerospermum* isolate TC09 for 10 days. (**h**) Growth of plantlets previously treated without (tray on left, control) and with (tray on right) volatile compounds (VCs) 1 month after transplanting to soil in 1020 trays. In this representative comparison, control tray contains 36 surviving plants out of 100 transplanted plants. The tray on right side has 46 surviving plants out of 52 transplanted plants. (**i**) Normal growth and development of in vitro propagated “Bailey-OP” plants 3 months after transplanting.

To address this bottleneck, we developed and tested an improved shoot proliferation system using many peach varieties including open-pollinated Bailey (*P. persica* “Bailey-OP”) (Figure 7 and Procedure S7). Using the established conditions, a typical single shoot tip can form a cluster with 50 to 100 individual shoots in 2 months (Figure 7b,c). We further incorporated the use of volatile compounds (VCs) of *Cladosporium sphaerospermum* strain TC09 to improve the otherwise previously reported long-term and laborious root induction process involving peach in vitro shoots [53,54]. As demonstrated in Figure 7, *C. sphaerospermum* dramatically enhanced root growth in “Bailey-OP” in vitro shoots. On average, up to 87% of VC-treated rooted shoots acclimatized successfully to the soil conditions and developed into robust plants as compared to 38% acclimatization rates among control shoots without VC treatment. Thus far, over 30 peach genotypes/varieties have been tested and many yielded similar rates of shoot proliferation.

**Figure 8:**
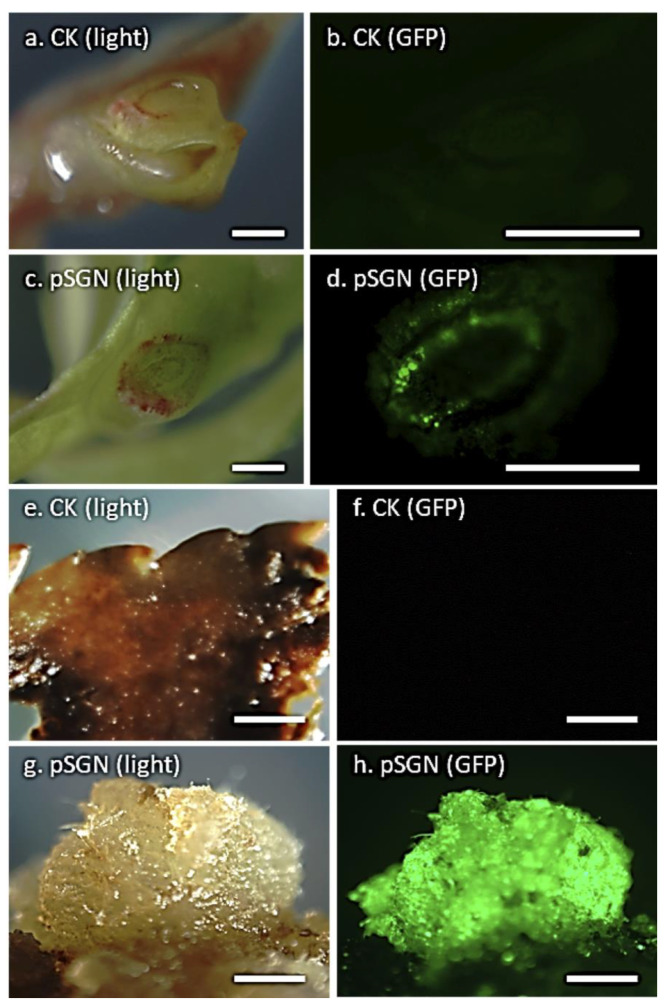
Transient and stable GFP expression detected in peach (*P. persica* cv. “Bailey-OP”) shoot explants transformed with the binary vector pSGN. Detection of transient GFP expression in non-transformed (white light (**a**) and UV light (**b**)) and transformed (white light (**c**) and UV light (**d**)) shoot explants 1 week after transformation. Detection of stable GFP expression in callus tissue derived from control (white light (**e**) and UV light (**f**)) and transformed (white light (**g**) and UV light (**h**)) shoot explants after 1 month in selection with 100 mg/L kanamycin (bar = 2 mm).

*Agrobacterium*-mediated genetic transformation experiments were conducted with the strains GV3101 and EHA105 harboring the pSGN binary plasmid [55], containing the enhanced *gfp* (*egfp)* and *npt*II marker genes. Shoot explants were prepared by carefully cutting across the apex region of axillary buds in each shoot of 2–3 cm in length to expose the meristematic tissues for *Agrobacterium* infection. Following transformation and 1-week cultivation, GFP expression was not detected in control explants without *Agrobacterium* exposure (Figure 8a,b). On the other hand, over 90% infected shoots showed transient GFP expression in one or more cut sites (Figure 8c,d). Although further microscopic examination needs to be conducted to provide proof, vascular tissues seemed to be more prone to infection, as indicated by the ring form of GFP-expressing cells across the infection site (Figure 8d). Noticeably, the lack of GFP expression in the meristematic dome region still needs further investigation. One month after culturing on kanamycin-containing (100 mg/L) shoot development medium (Procedure S7), no callus growth was found in any control explants (Figure 8e,f), while GFP-stable expressing calli developed from *Agrobacterium*-infected explants (Figure 8g,h). Putative transgenic shoots were also recovered. However, GFP expression was not detected in leaves of shoots that survived 3 to 4 months of kanamycin selection (100 mg/L). This indicated that the recovered shoots were non-transgenic escapes. The main reason could be the significantly reduced selection pressure on target cells due to the filtering effect of the relatively large-size parental stem section. The growing shoots may also have developed from un-exposed, pre-existing shoot primordia around the cut site. On the other hand, we tested excised individual axillary buds, with or without slicing through the middle region, using similar transformation conditions, and only recovered non-organogenic transgenic calli at low frequencies. Quite often, the majority of these excised small explants quickly turned necrotic and succumbed to *Agrobacterium* overgrowth.

##### Nodal Explants

Following Procedure S8, in vitro “Bailey-OP” shoots were used as the source of nodal explants. For the transformation experiments, the disarmed *A. tumefaciens* EHA101 strain harboring the binary plasmid pVNFbin was used.

**Figure 9:**
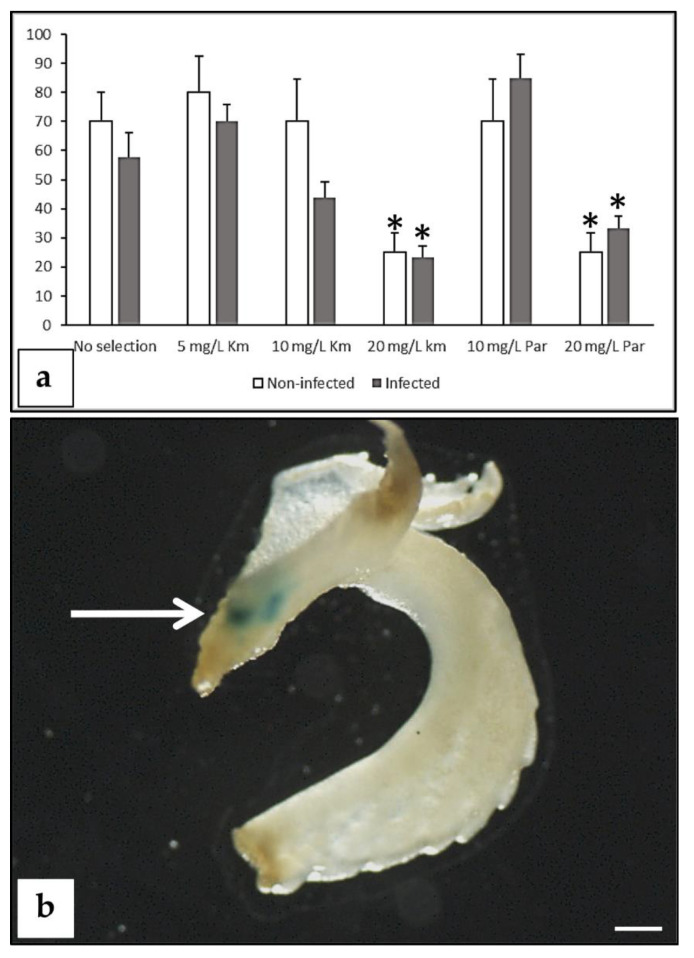
Regeneration and transformation from peach nodal explants. (**a**) Effect of antibiotics on adventitious regeneration from non-infected explants and EHA101 (pVNFbin)-infected explants. Regeneration data were collected at 6 weeks from the beginning of the experiment. Asterisks indicate statistical significance (*p* < 0.01) compared to the “no selection” treatment according to Pearson’s chi-test. A total of 565 explant “Bailey-OP” were used for this study. Experiment was repeated at least twice. (**b**) Chimerical regenerated shoot showing GUS activity (arrow) (bar = 1 mm).

The addition of 20 mg/L kanamycin or 20 mg/L paromomycin to the regeneration medium significantly (*p* < 0.01) reduced regeneration compared to the controls without the addition of antibiotics (Figure 9a). Statistical differences between regeneration of non-infected and infected explants within the same treatment were not found for any of the different selection strengths applied (Figure 9a). After 6 weeks from the beginning of the experiment, all green, healthy buds were isolated and placed onto a meristem development medium [56] supplemented with 15 mg/L kanamycin. They were subcultured onto fresh medium every 2 weeks. All buds regenerated from non-infected explants became chlorotic and died in 2–4 weeks. Some of the buds regenerated from the infected explants were able to survive longer during the selection process. Surviving buds were subcultured, and transformation evaluation was conducted by GUS assays and/or molecular tests (PCRs or Southern blots). On the basis of GUS assays, two chimeras were detected, as the blue staining was only located in a particular area of the bud (Figure 9b). Molecular tests revealed that none of the surviving shoots by the end of the selection procedure were transgenic. The two chimerical shoots originated from the experiment where 20 mg/L paromomycin was applied for selection, reaching 1.7% transformation efficiency. On the basis of these results, it seems that the selection applied was too low, since non-transgenic escapes and chimeras survived. Further studies should be conducted with more stringent selective conditions or applying a gradual increasing selection to be able to dissociate chimeras and recover completely transformed shoots.

##### Meristematic Bulks

An adventitious shoot regeneration method, based on the generation of a meristematic bulk (MB) from shoot tips, has been applied successfully in different peach cultivars (“Big top”, “Zaitabo”, “UFO-3”, “Maruja”, “Flariba”, and “Alice Bigi”) and in *P. persica x P. amygdalus* rootstocks (“GF677”, “Garnem”, and “Hansen 536”) [14,20,57,58]. Furthermore, this method allowed the regeneration of transgenic plants of the peach-almond hybrid “GF677” [14]. A similar protocol has been applied to other perennial plant species, such as grapevine and blueberry [59,60], showing its versatility and potential for in vitro regeneration and/or genetic transformation of fruit species.

To improve the previously described procedure in peach [14], two factors that may affect *Agrobacterium*-mediated transformation were further studied: (i) the addition of phenolic compounds such as acetosyringone (AS), and (ii) the utilization of ethylene inhibitors such as STS. The addition of AS increased *Agrobacterium*-mediated transformation in apricot [26,61] and almond [62,63]. Furthermore, endogenous plant level of ethylene reduces *Agrobacterium*’s ability to transfer the T-DNA into plant cells [64].

Transformation experiments were performed in the peach x almond hybrid rootstock “Garnem” and the peach cultivars “UFO-3” and “Alice Bigi”. Two disarmed *A. tumefaciens* strains C58 (pMP90) [65] and EHA105 [66], both carrying the binary plasmid pBin19-*sgfp* [67], were utilized. AS was added to the bacterium culture medium and transgenic cells were selected with 50 mg/L kanamycin. Transformation was monitored through GFP expression. Two different explants were used for transformation: (1) the basal part of the shoots, which would produce the MB, and (2) slices of the MB. In both cases, GFP was only expressed transiently, and stable transformation was not detected. A pre-culture in darkness after infection enhanced the number of cells showing GFP signal and the stability of them (data not shown). In this study, the effect of the addition of AS to the bacterium medium was genotype dependent; it produced no effect in “Garnem”, was counterproductive in the case of “Alice Bigi”, and generated different results depending on the *Agrobacterium* strain in “UFO-3” (not shown).

**Figure 10:**
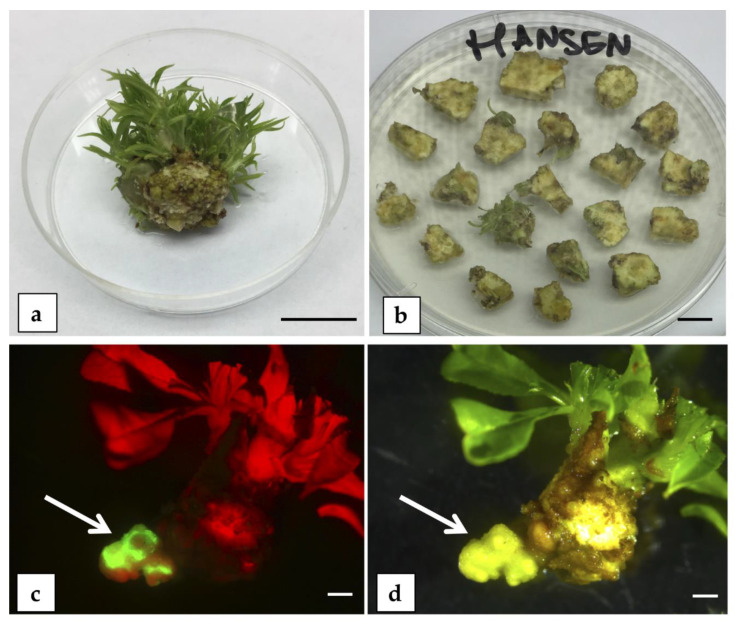
Organogenesis trials on peach meristematic bulks (MBs). (**a**) MB of “Hansen 536” (bar = 1 cm). (**b**) Slices (1 cm^2^, 2 mm thick) obtained from “Hansen 536” MBs used as starting explants for *A. tumefaciens*-mediated transformation trials (bar = 1 cm). “Hansen 536” stably transformed callus-expressing eGFP (arrow) observed under UV light (**c**) or under white light (**d**)**.** Photographs taken at 3 months post-infection (bar = 2 mm).

Additional trials were performed to improve the previous transformation results obtained in “Hansen 536” MBs [20]. The two factors studied were (i) the addition of AS in the co-culture medium at concentrations of 0, 50, 100, and 200 µM, and (ii) STS added during the co-culture period and/or in the regeneration/selection medium for the first 2 weeks after *Agrobacterium* infection. Transformation trials were carried out following the protocol described by Sabbadini et al. [20] with MB slices used as starting explants (Figure 10a,b) and the EHA105 *A. tumefaciens* strain harboring a construct with the *nptII* and *egfp* genes. The addition of AS, at the concentrations tested, or the different STS treatments assayed did not influence the transformation efficiency at 3 months post-infection compared to the controls. From these experiments, transformed shoots were not obtained and only portions of stably transformed callus expressing *egfp* were observed (Figure 10c,d), similar to previously obtained results [20].

## 4. Possible Solutions to the Bottleneck

In order to produce transgenic plants successfully, there should be an overlap between peach tissue cells able to regenerate adventitious shoots and those amenable for transformation. In general, the lack of an efficient adventitious regeneration protocol is the limiting factor for gene transfer technologies in fruit tree species. The results presented in this manuscript, together with previously published studies, show that adventitious shoot regeneration, while genotype-dependent, does not seem to be the major problem for this species. Both the integration of the transgene/s into the plant genome and then the recovery of uniformly transformed plants are problematic. Even with regeneration rates as high as 40–100%, the production of the transgenic shoots has either not been possible or has been successful only with an extremely low efficiency, therein frequently producing shoots that are chimeric for transformation. Considering the extremely low rate of transformation (zero most of the time) and the regeneration of chimeric shoots as the main bottleneck to genetic engineering of peach, this section discusses different possibilities to improve *Agrobacterium*-mediated transformation and selection of transformed tissues of peach.

In plant transformation, an appropriate selection protocol is essential to obtain transgenic plants. Most of the peach transformation protocols published include aminoglycoside antibiotics for selection. It is known that this class of antibiotics can interact with both the membrane of cells and their receptors and with components of the culture medium (Ca^2+^). Their activity is also affected by light, pH, and/or temperature [68]. Antibiotic concentration decreases in the medium due to degradation in the vicinity of transgenic cells capable of inactivating them [69], and thus it has been frequently suggested as a subculture to avoid escapes and chimeras. Researchers should take into account all these particularities of antibiotics to apply the most appropriate concentrations to plant tissues at each step of development. In peach, it would be important to determine which concentrations are limiting the growth of the initial explant, as well as the initiation and development of meristems and shoots, in order to establish a selection protocol coupled to organogenesis and organ development. The use of alternative selective marker genes could also be part of the solution. In peach, we have tried the *bar* and *ipt* genes in mature seed hypocotyl sections without success. However, these selective marker genes could be appropriate for other peach explants. Moreover, selection strategies based on mannose or hygromycin have shown to be amenable for other *Prunus* spp. [29,30,70,71]. Further studies that are focused on selection methods should be carried out, including combining visual marker genes, such as *GFP*, and histological studies to verify the different competences of cells in transformation and regeneration processes.

New studies on genetic factors determining peach cell transformation recalcitrance could help to find molecular solutions to develop efficient protocols. During the past 45 years, model plant systems, such as *Arabidopsis,* have been exploited to describe and understand the T-DNA transfer and insertion into the host plant genome at a molecular level, due to their ease of expressing the transgene, both in transient and stable transformation. From our perspective, studies reporting the reduction of transient and/or stable transformation efficiency in some *Arabidopsis* ecotypes are of particular interest, including examples of recalcitrance to *Agrobacterium* transformation (reviewed by [72]). Collectively, these reports showed how the *Agrobacterium*-mediated gene delivery system in plants could fail at any step of the process, including the first physical contact of bacterium to plant tissue, delivery of T-DNA from the bacterial cytoplasm up to its importation, or integration and expression in the plant nucleus. It is interesting to note that peach trees are quite susceptible to crown gall [73]. Hwang et al. [72] reviewed the role of several plant key genes participating in all these events, indicating the possibility that their focused over-expression and/or downregulation enhanced transformation rates of both recalcitrant and susceptible model plant lines. We suggest that this approach may be promising and suitable to almost all the species of the genus *Prunus*, which are (with the exception of plum) recalcitrant to *Agrobacterium.* Genes affecting plant regeneration itself could be useful in the road to improve peach genetic transformation; an example of this is the ectopic expression of the corn meristem identity gene *KNOX*1 in plum plants, which significantly improved adventitious shoot regeneration from plum leaf explants [74].

The *Agrobacterium*–host interaction is a war of cell survival, in which the host defense system combats the intruding pathogen. As suggested by a pioneering work carried out by Dunoyer et al. [75], plant defense reactions rely on the induction of RNA silencing pathways to limit the expression of bacterial T-DNA. Therefore, to enhance peach competence for *Agrobacterium*-mediated transformation, alternative strategies may consist in attenuating the reaction of plant defense responses in infected tissues. As master gene silencing regulators, microRNAs are involved in many developmental processes, such as organogenesis, somatic embryogenesis, and resistance against pathogens [76,77]. Some years ago, researches were committed to build microRNAs libraries with the main aim of evaluating how these molecules alter their expression profiles during in vitro developmental stages. These results are crucial for the optimization of more suitable in vitro culture conditions, especially for recalcitrant species. In particular, several studies reporting microRNA expression patterns, both from model plant and some cultivated crop tissues, during different bacterial infections showed that bacterial elements trigger the up- or downregulation of specific microRNAs, which suppress or induce key negative or positive regulators of the host defense (reviewed by [78]). In addition, key microRNAs involved in somatic embryogenesis [79] suggest that increasing our understanding about the role of these molecules could also contribute to improved gene transfer protocols based on SE. In peach, several microRNAs involved in response to different stress conditions have been identified [25,80,81,82,83,84]; nevertheless, microRNA expression profiles from *Agrobacterium*-mediated transformed or infected tissues has not been built to date. Gaining an understanding of the role of microRNAs and their target mRNAs in preventing genome modification may be useful in elucidating appropriate in vitro stimuli capable of inducing efficient *Agrobacterium*-mediated transformation in recalcitrant in vitro cultures, including peach. Moreover, the addition of antioxidants to cope with toxicity of reactive oxygen species (ROS) generated as a result of the *Agrobacterium* infection may improve peach transformation as has been described in other plant species such as Mexican lime and tomato [85,86].

Lastly, following its first detection in the middle of the 20th century [87,88], *A. rhizogenes*-mediated adventitious hairy root disease in dicotyledonous plants has been widely investigated and used as a transgenic tissue generation system in plant biotechnology, mainly as an alternative option for *A. tumefaciens* gene delivery in plants [72]. As reviewed by Giri and Narasu [89], adventitious shoot regeneration can occur directly from transgenic roots or by moving them to regeneration medium. As recently shown, the transgenic hairy root phenotype has been induced in different peach explants such as leaves, hypocotyls, and shoots using *A. rhizogenes* strain MSU440 [22]. The main goal of this study was to optimize a reproducible *A. rhizogenes*-mediated transformation protocol for gene function and genetic engineering studies in peach. Although adventitious regeneration from in vitro root cultures is difficult, an efficient shoot regeneration method from roots in *P. persica* could be a further approach for peach genetic improvement, as the production of peach transgenic plants through *A. tumefaciens* has been arduous to date.

The utilization of novel DNA delivery methods in peach should be further studied. In recent years, nanoparticles have been extensively utilized in many areas of research (reviewed by [90]). Successful nanoparticle-mediated introduction of DNA plasmids into plant cells at relatively high efficiencies has been demonstrated (e.g., [91,92]). The methodology is relatively simple and may offer certain advantages such as the absence of phytotoxicity and high target cell coverage.

## 5. Conclusions

Many regeneration protocols are available from different type of peach tissues, some of them demonstrating a high efficiency. Nevertheless, regeneration has not led to the reliable production of uniformly transgenic peach plants. In general, with regeneration approaches that involve adventitious organogenesis, the main issue remains the selection procedure for obtaining non-chimeric regenerated shoots, while the limit of SE is the development of efficient regeneration protocols, in particular from adult tissues. Peach immature cotyledons allowed efficient shoot regeneration through organogenesis and SE, but with low transformation rates.

Protocols for the development of transgenic peach cultivars are needed to apply new biotechnological tools that can help to resolve important problems affecting peach cultivation, in order to increase sustainability, resilience, and quality. Future and current fruit tree breeding programs should integrate classical- and biotechnologies. For the development of a reliable peach transformation system, the key issues to be researched are the low efficiency of *A. tumefaciens*-mediated transformation, the low level of correspondence between cells competent for transformation and those that have regeneration competence, and the high rate of chimerism in the few shoots that are produced following transformation procedures.

While we currently have focused on the scientific aspects of developing improved peach cultivars through genetic engineering, a major impediment to the application of this and other novel genetic technologies in applied fruit breeding is, in general, the lack of clear, efficient, and science-based regulatory regimes.

## Figures and Tables

**Table 1:** Transformation in *Prunus persica* L.

Genotype	Method (Strain)	Plasmid (Genes)	Explant	T.E. ^a^ (%)	Main Advantage	Main Disadvantage	Reference
“14DR60”	*A. tumefaciens* (A281)	pGA472 (*npt*II)	Embryogenic callus, leaves, and immature embryos	0	All three starting explants developed calli, which were able to grow in a medium containing the selective agents.	Typically, long-term embryogenic peach cultures produce few normal shoots.	Scorza et al. [15]
“Tennessee natural”							
“PER 2D”							
“Redhaven”	*A. tumefaciens* (tms328::Tn5)	pTiA6 (*iaa*, *ipt*)	Shoots	0	Demonstration of potential for using *A. tumefaciens* to transfer genes to peach.	Shoots could not be regenerated from the transformed cells.	Hammerschlag et al. [16]
			Immature embryo axes	n.s.	Demonstration of regeneration of plants from embryo-derived callus infected with the shooty mutant strain of *A. tumefaciens*.	Not reproduced in other laboratories.	Smigocki and Hammerschlag [5]
“Lovell”	Biolistic	pBI505, pBI426 (*npt*II, *gus*)	Embryo calli, immature embryos, cotyledons, leaves, and shoot tips	0	Optimization of biolistic parameters for this species.	Unsuccessful recovery of plants from the transformed embryogenic calli.	Ye et al. [17]
“Miraflores”	*A. tumefaciens* (C58C1/pMP90)	pBin19-sgfp (*npt*II, *gfp*)	Mature embryo axes	3.6	Mature seeds are available year-round.	Not reproduced in other laboratories.	Pérez-Clemente et al. [12]
“Bailey”	*A. tumefaciens* (LBA4404, EHA105, GV3101, CG937, CG1052, CG1059)	pLC101 (*npt*II, *gfp*)	Cotyledons, embryonic axis, hypocotyl slices, callus, internodes, and leaves	0	Comprehensive evaluation of factors affecting *A. tumefaciens*-mediated peach transformation. Seed-derived internodes showed the highest transformation percentage compared to the other explants.	Rates of *GFP* transformation under the experimental conditions were low.	Padilla et al. [18]
“Lady Nancy”							
“Harrow Beauty”							
“KV930465”	*A. tumefaciens* (LBA4404, EHA105)	pBin19 (*npt*II, *gus*)					
“KV930408”							
“KV930303”							
“KV939455”	*A. tumefaciens* (LBA4404)	pBISNI, pGA482Ggi (*npt*II, *gus*)					
“KV930478”							
“KV930311”							
“Akatsuki”	Electroporation	pBI221, pE2113-GUS, PL-GUS (*gus*)	Protoplasts from immature fruits mesocarp	0	The system can be applied for expression analysis of genes isolated from other *Rosaceae* species.	The period suitable for protoplast isolation is limited to about 1 week.	Honda and Moriguchi [19]
“O’Henry”	*A. tumefaciens* (GV3101, EHA105)	pBIN-m-gfp5-ER (*npt*II, *gfp*)	Immature cotyledons	0.6	Very efficient regeneration protocol.	Explants available for only a limited time each year (50 to 70 days post-bloom). Not reproduced in other laboratories.	Prieto [13]
“Rich Lady”							
“GF677” ^b^	*A. tumefaciens* (GV2206)	hp-pBin19 (*npt*II)	Meristematic bulks	0.3	The first successful report of a peach rootstock genetic transformation using adult tissue as starting material.	The efficiency of the procedure was relatively poor.	Sabbadini et al. [14]
“Hansen 536” ^b^	*A. tumefaciens* (EHA105)	pK7WG2-ihp35S-PPV194::eGFP (*npt*II, *gfp*, *PPV* polyprotein hairpin)	Meristematic bulks	0	Uses adult tissues as source of explants.	Shoot regeneration from transgenic calli was not obtained.	Sabbadini et al. [20]
	*A. tumefaciens* (EHA105, LBA4404, GV3101)	pBISN1 (*npt*II, *gus*)	Leaves	0	Adult tissue available year-round.	Only transient transformation was recorded.	Zong et al. [21]
“Shantao”	*A. rhizogenes* (MSU440)	pMV2G + Ri Plasmid (*DsRED1*) + (*rol* genes)	Leaves, hypocotyls, and shoots	27.8 ^c^	This protocol provides a way to evaluate gene functions, genetic engineering, and root-rhizosphere microorganism interaction in peach.	Only transgenic hairy roots were regenerated. Transgenic shoots were not produced.	Xu et al. [22]
“Shengli”				50.9 ^c^			
“Lvhuajiuhao”				30.7 ^c^			
“Shengli”		pSAK277 (*PpMYB10.1*)	Shoots	n.s. ^c^			

^a^ Transformation efficiency (number of transgenic shoots obtained per 100 explants). When not indicated, it was not specified (n.s.) by authors. ^b^ *Prunus persica* x *Prunus* *amygdalus* hybrids. ^c^ Efficiency of regeneration of transgenic hairy roots.

**Table 2:** Further approaches applied to improve *P. persica* L. regeneration–transformation.

Genotype	Explant	Route of Morphogenesis	Shoot Regeneration Rate ^a^ (%)	Transformation Method	Plasmid (Genes)	Selection Strategy (Agent)	Outcome/Comments	Main Advantage	Main Disadvantage
**Assessed methodologies that involve peach juvenile tissues**									
“O’Henry”, “Elegant Lady”, “Rich Lady”, “Venus”	Immature cotyledon	Somatic embryogenesis	60.0	Not assayed ^d^	--	--	A similar regeneration procedure coupled with *A. tumefaciens* has previously succeed in the generation of transgenic peach plants [13].	Consistent whole plant production.	Explants available for only a limited time each year (50 to 70 days post-bloom).
“Bailey”, “Guardian”, “Starlite”		Organogenesis	80.0	*A. tumefaciens* GV3101	pVNFbin (*nptII, gus*)	Negative: early or late (kanamycin)	Selection failed. All surviving shoots were escapes.	Efficient adventitious regeneration.	Explants available for only a limited time each year (45 to 50 days post-bloom).
“TruGold”	Mature hypocotyl slice	Organogenesis	14.0	*A. tumefaciens* (EHA101, GV3101)	pVNFbin (*nptII, gus*)	Negative: early (paromomycin)	Selection failed. All surviving shoots were escapes.	Explants available year-round (mature seeds can be stored at 4 °C for several years).	Plants regenerated from transformed tissues via organogenesis may be chimeras.
“Bailey”			32.0	*A. tumefaciens* EHA105	*ipt*-containing construct (*ipt, gus*)	Positive: early (low-level TDZ)	Selection failed. All regenerated shoots were escapes.		
“Nemaguard”			43.0	*A. tumefaciens* EHA105	pBarGUS (*bar, gus*)	Negative: early (BASTA)	Selection failed. All surviving shoots were escapes.		
“Bailey”	Seed-derived internode	Organogenesis	42.9	*A. tumefaciens* EHA101	pVNFbin (*nptII, gus*)	Positive: early (paromomycin)	All shoots died during selection.	Explants available year-round (mature seeds can be stored at 4 °C for several years).	Plants regenerated from transformed tissues via organogenesis may be chimeras.
**Assessed methodologies that involve peach adult tissues (cultivars or rootstocks)**									
“Hansen 536” ^b^	Leaf from meristematic bulk	Somatic embryogenesis	0	Not assayed	--	--	--	Explants available year-round.	Potential for somaclonal variation due to the use of a highly differentiated tissue such as leaf.
“GF677” ^b^	Petal and anther		0			--		Regeneration via SE reduces the formation of chimeras.	Explants must be tested at different developmental stages, which can influence SE.
“EVD 1”, “EVD 2”, “EVD 3”, “EVD 44288”, “Redglobe”, “Redhaven”, “Coacalco-OP ^c^”, “Rutgers Redleaf-OP”, “Sihung Chui Mi-OP”, “Nemaguard-OP”, “Indian Cling OP”	Leaf	Organogenesis	0 (only root regeneration observed)	Not assayed ^d^	--	--	--	Explants available year-round.	Inefficient adventitious shoot regeneration protocol.
“Bailey-OP”	Axillary shoot	Organogenesis	100.0	*A. tumefaciens* (GV3101, EHA105)	pSGN (*nptII, eGFP*)	Negative: early (kanamycin)	Selection failed. All surviving shoots were escapes.	Consistent whole plant production.	Plants regenerated from transformed tissues via organogenesis may be chimeras.
	Nodal explant	Organogenesis	73.3	*A. tumefaciens* EHA101	pVNFbin (*nptII, gus*)	Negative: early (paromomycin)	Two chimerical shoot lines detected (1.7% transformation efficiency).	Explants available year-round.	Plants regenerated from transformed tissues via organogenesis may be chimeras.
“UFO-3′, “Alice Bigi”, “Garnem” ^b^	Meristematic bulk	Organogenesis	25.0, 8.3, 91.6 (respectively)	*A. tumefaciens* (C58, EHA105)	pBin19-sgfp (*nptII, GFP*)	Negative early (kanamycin)	Transient transformation.	The explants are produced in a relatively short period of time (90 days).	Certain probability of somaclonal variation induced by increasing concentrations of cytokinins applied to the initial explant.
“Hansen 53”′ ^b^	Meristematic bulk	Organogenesis	80	*A. tumefaciens* EHA105	pK7WG2-ihp35S-PPV194::eGFP (*npt*II, *gfp*, *PPV polyprotein* hairpin)	Negative: early or late (kanamycin)	Stably transformed calli.		

^a^ Shoot regeneration rate under non-selective conditions. ^b^ *P. persica* x *P.* *amygdalus* hybrids. ^c^ OP: Open-pollinated. ^d^ Transformation experiments were not performed in this case.

**Table 3:** Effect of thidiazuron (TDZ) concentration on peach (cv. “Bailey”) mature seed hypocotyl section organogenesis: comparison among non-infected and infected explants with *A. tumefaciens* EHA105 strain harboring an *ipt*-containing construct.

TDZ (µM)	Treatment	Explants	Shoot Regeneration (%)	Root Regeneration (%)
0	control	70	2.9	85.7
	*ipt*-infected	39	7.7	38.5
2.5	control	80	10.0	20.0
	*ipt*-infected	65	7.7	7.7
5.0	control	104	21.1	26.0
	*ipt*-infected	350	12.3	2.3
7.5	control	74	12.2	8.1
	*ipt*-infected	79	15.2	3.8

Experiments were repeated at least twice.

**Table 4:** Organogenesis from germinated peach seeds internodes (cv. “Bailey”).

Citokinin Added to the Regeneration Medium	Seed Germination					
	Without BAP			40 µM BAP		
BAP (μM)	Regeneration (%)	Shoots/Explant	Roots (%)	Regeneration (%)	Shoots/Explant	Roots (%)
**0**	3.7 ^c^	1	0	0 ^c^	0	40
**1**	10 ^b,c^	1.3	0	0 ^c^	0	18
**5**	30.6 ^a^	1	0	19.1 ^b^	1	0
**10**	39.3 ^a^	1.7	0	42.9 ^a^	1.7	0

Data were collected after 8 weeks from the beginning of the experiment. Experiment was repeated three times with 10 explants per treatment. Different letters indicate statistical significance (*p* < 0.05) according to chi-square test.

**Table 5:** Adventitious rooting from leaf segments of greenhouse-grown bud-grafted plants and in vitro-grown peach seedlings.

Genotype	Rooting (%)		Average Root Number	
	Dark	Light	Dark	Light
**Greenhouse-grown ^a^**				
“EVD 1”	40	5	6.5	2.7
“EVD 2”	35	0	3.7	0
“EVD 3”	47	0	2.3	0
“EVD 44288”	61	0	2.9	0
“Redglobe”	60	0	2.3	0
“Redhaven”	44	0	2.1	0
**In vitro-grown ^b^**				
“Coacalco OP”	92	35	7.0	2.6
“Rutgers Redleaf double haploid OP”	58	9	4.9	2.0
“Sihung Chui Mi OP”	73	19	5.1	4.1
“Nemaguard OP”	84	25	6.6	3.4
“Indian Cling OP”	90	6	5.3	1.0

^a^ Within greenhouse-grown leaves, ANOVA indicated that light was a significant factor at *p* = 0.0001 for both percentage rooting and number of roots. ^b^ Within in vitro-grown leaves, ANOVA indicated that light was a significant factor for percentage rooting at *p* = 0.0001 and for number of roots at *p* = 0.012.

## Supplementary Materials

The following are available online at https://www.mdpi.com/2223-7747/9/8/971/s1, Figure S1: Somatic embryogenesis (SE) in peach from immature cotyledons. Procedure S1: Adventitious bud regeneration from peach immature cotyledons. Procedure S2: Adventitious bud regeneration from peach mature hypocotyl sections. Procedure S3: Adventitious bud regeneration from seed-derived internodes. Procedure S4: SE from in vitro leaf explants. Table S1. Different concentrations and combinations of PGRs and amino acids used in leaf SE induction media. Procedure S5. SE from peach petals and anthers. Procedure S6: In vitro organogenesis of roots from peach leaf explants. Procedure S7: Improved peach in vitro shoot proliferation. Procedure S8: Bud regeneration from in vitro peach nodal explants.
