# Supplementary material for: Genetic Transformation in Peach (Prunus persica L.): Challenges and Ways Forward
Source: Plants (Basel). 2020 Jul 31;9(8):971. doi: 10.3390/plants9080971 (PMC7465125; doi:10.3390/plants9080971)
Supplement: Supplementary file 1 [file plants-09-00971-s001.pdf]

Supplementary Material

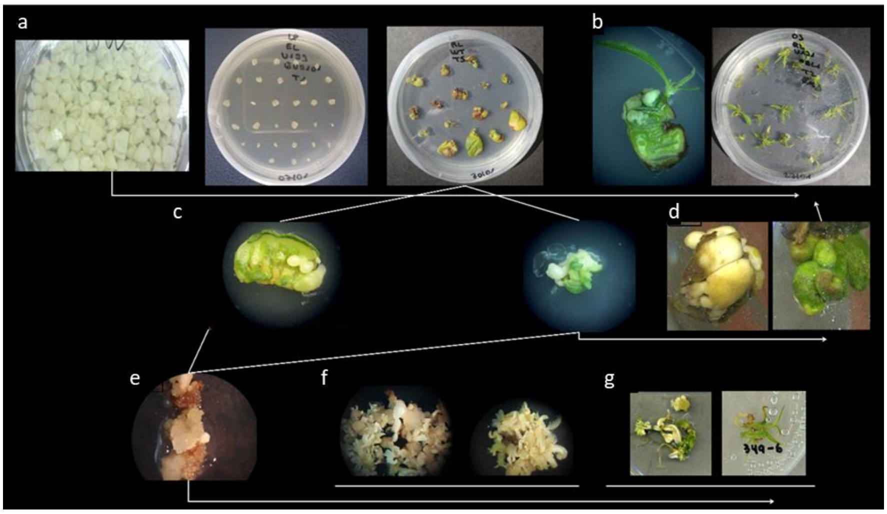

Figure S1. Somatic embryogenesis (SE) in peach from immature cotyledons. In a regular procedure designed for direct organogenesis, immature cotyledons obtained 50–70 d post bloom **(a)** are led to direct budding in LP modified medium [1] **(b)** with 6-benzylaminopurine (BAP) (5.0 μM) and α-naphthaleneacetic acid (NAA) (between 3.0 and 5.0 μM) in a process that requires between 3 and 6 months. This treatment will lead to the formation of white-egg shape structures **(c)** that can be induced to organogenesis by additional culturing for 30-60 d in LP medium [1] supplemented with 1.0 μM 2,4-dichlorophenoxyacetic acid (2,4-D) **(d)**. In the process, callus formation **(e)** will be also obtained whose lead to continuous somatic embryo formation **(f)** by culturing these explants in LP medium supplemented with sucrose (4%). This latter procedure **(e + f)** can be kept for at least one year. Although genetic transformation was not evaluated, this procedure allowed whole plant production **(g)**. All the procedures are carried out under a 16 h light photoperiod at 22 ± 1 °C.

1. Procedure SM1: Adventitious Bud Regeneration from Peach Immature Cotyledons.

Immature fruits of 45-50 days after blooming are collected and surface disinfected with 70% (*v*/*v*) ethanol. Immature cotyledons are taken from the seeds in a laminar hood and placed on Preculture Medium with the adaxial face down for 7 days in the dark at 24 ± 1 °C. Preculture medium consists of Quoirin and Lepoivre (QL) salts and vitamins [2], 3% sucrose, 5 μM 2,4-D, 0.5 μM BAP and 0.65% bactoagar. Following the preculture, explants are placed on regeneration medium QL salts and vitamins [2], 3% sucrose, 12.5 μM thidiazuron (TDZ), 0.4 μM NAA, 60 μM silver thiosulphate (STS), 0.7% bactoagar] and transferred to a 16/8 h light/dark cycle, light intensity of 45–50 μE $m^{-2}s^{-1}$, and a temperature of 24 ± 1 °C. Adventitious buds start appearing as direct regeneration after 4–5 weeks, normally as cluster throughout the whole adaxial surface of the explant (Figure 1 a, b, c).

As buds/clusters appear, follow a two-step procedure previously described [3] to rescue and elongate them. Briefly, clusters/buds are firstly cultured on a meristem development medium consisting of QL salts and vitamins [2], 2% sorbitol, 7.09 μM BAP, 0.05 μM indole-3-butyric acid (IBA) and 0.7% bactoagar. After 2-4 weeks, buds/clusters have grown up as a rosette of 0.5–1.0 cm wide. At this point, rosettes are transferred to shoot elongation medium consisting of QL salts and vitamins, 2% sorbitol, 3.5 μM BAP, 5.8 μM gibberellic acid (GA_3), 0.05 μM IBA and 0.7% bactoagar. As shoots elongate from the rosette and reached 2-3 cm long, they are excised and placed in rooting medium previously designed for apricot [4]. Rooting medium consists of QL macros [2], DKW micros and vitamins [5], 2% sucrose, 1.5 mM $CaCl_2$, 0.8 mM phloroglucinol, 2.95 μM IBA, 29.6 μM adenine and 0.7% bactoagar. Adventitious roots start being visible after two weeks of culture in rooting medium and with two more additional weeks, shoots show a well-developed root system (Figure 1 d). All media pHs are adjusted to 5.7 and sterilized in an autoclave at 121°C for 20 min. Acclimatization of shoots is accomplished following standard procedures (Figure 1 e) [6].

2. Procedure SM2: Adventitious Bud Regeneration from Peach Mature Hypocotyl Sections.

Mature seeds stored at 4 °C are used. Seeds can be stored for several years. For seeds disinfection, the endocarp is removed with a nutcracker, and seeds are immersed in a beaker containing a solution of 20% commercial bleach (commercial bleach = 6.15% sodium hypochlorite) and 0.02% Tween-20 for 20 min and rinsed three times with sterile water in a laminar flow bench. Disinfected seeds are soaked in sterile water overnight at 4 °C. The seed coat is removed with the aid of a scalpel, the radicle and the epicotyl are discarded, and the hypocotyl is sliced into 2–4 cross sections (0.5–1 mm), which are placed on the regeneration medium. Regeneration medium consists of QL basal salts [2], MS vitamins [7], 2% (*v/v*) sucrose, 7.5 μM TDZ, 0.25 μM IBA, 0.5 μM aminoethoxyvinylglycine (AVG) and 0.7% (*v/v*) purified agar (Sigma). The pH is adjusted to 5.8 before autoclaving at 121°C for 20 minutes. Explants are cultured in dark conditions for 2 weeks before transferring them to a 16/8 h light/dark photoperiod, light intensity of 45–50 μE m^{-2} s^{-1}, and a temperature of 24 ± 1 °C. First buds start appearing after 4 weeks from the beginning of the experiment as direct adventitious regeneration, and additional buds appeared in the 2–3 following weeks. As buds/clusters appear, follow the two-step procedure described in Procedure SM1.

3. Procedure SM3: Adventitious Bud Regeneration from Seed-Derived Internodes.

Seeds are induced to germinate without cold stratification by soaking overnight in a 1:1 solution of 500 ppm BAP and 500 ppm GA_3 and, subsequently, germinated in vitro in the dark on C2D medium [8] with the addition 40.0 μM BAP, 5.0 μM TDZ, 5.0 μM IBA, MS vitamins [7], 100 mg/L myoinositol, 3% sucrose and 0.6% Bacto agar. Upon germination (12 to 17 days), the internode from the basal section of the germinating epicotyl immediately above the point of attachment with the cotyledon is sliced into 2–3 mm sections and used as primary explant. The regeneration medium consists of LP macro and micronutrients (LP1) [9], MS vitamins [7], 2.5% sucrose and 100 mg/L myoinositol and 0.6% Bacto agar. Medium is supplemented with 1.0 μM NAA and 10.0 μM BAP. All media pHs are adjusted to 5.8 and sterilized in an autoclave at 121 °C for 20 min.

4. Procedure SM4: SE from in vitro Leaf Explants.

Leaves are cultured with the abaxial side in contact with the SE induction media. The induction media consisted on Murashige and Skoog (MS) basal salts and vitamins [7], 20 g/L sucrose, and 7 g/L plant agar (Duchefa Biochemie, Italy), pH 5.7–5.8, supplemented with several combinations of plant growth regulators (PGRs) and amino acids (Table SM1). Leaves were incubated in darkness at 24 ± 1 °C for seven weeks and then exposed to light (16-h photoperiod at a light intensity of 70 μE $m^{-2}s^{-1}$) at 24 ± 1 °C for five weeks.

Explants placed on media A, B and E (Table SM1) produce cream-colored calli after 10-12 weeks of culture. These calli are then transferred to a PGRs-free medium consisting of MS basal salts and vitamins [7], 20 g/L sucrose, 7 g/L plant agar (Duchefa Biochemie, Italy), and 0.5 g/L activated charcoal (Duchefa Biochemie, Italy), pH 5.7–5.8. Cultures are maintained under the light and temperature regime described above.

Table 1. Different concentrations and combinations of plant growth regulators (PGRs) and amino acids used in leaves somatic embryogenesis (SE) induction media.

SE Induction Media	PGRs and Amino Acids (μM)					
	BAP	NOA	NAA	2,4-D	GA_3	Proline (mM)
A	2.2	0.5				
B	8.9		10.7			
C	2.2	0.5			0.4	2.6
D	4.4		0.3		0.4	2.6
E				14		2.6

5. Procedure SM5. SE from Peach Petals and Anthers.

Petals and anthers, from sterile unopened flowers, with filaments intact are carefully removed and cultured with the adaxial side (for the petals) in contact with the SE induction medium, whereas, anthers are horizontally placed on the medium. The induction medium for petals and anthers (PAM) consists of MS basal salts and vitamins [7], 20 g/L sucrose, and 7 g/L plant agar (Duchefa Biochemie, Italy), pH 5.7–5.8, supplemented with 14.0 μM 2,4-D and 0.22 μM BAP. Both explants are then maintained in darkness at 24 ± 1 °C for 5–7 weeks before transfer them to light (16 h photoperiod at a light intensity of 70 μE $m^{-2}s^{-1}$) at 24 ± 1 °C during 5 additional weeks.

Additionally, two media, named as PIV [10] and MSI [11], used for embryogenic culture induction from *Vitis* spp. anthers, were tested for 'GF677' anthers. PIV medium [10] consisted of Nitsch and Nitsch basal salts [12] and B5 vitamins [13], 60 g/L sucrose, and 3 g/L Phytagel (Sigma-Aldrich), pH 5.7 supplemented with 8.9 μM BAP and 4.5 μM 2,4-D. MSI medium [14] containing MS basal salts and vitamins [7], 0.1 g/L myo-inositol, 20 g/L sucrose, and 7 g/L plant agar (Duchefa Biochemie, Italy), pH 5.8 supplemented with 4.5 μM BAP and 5 μM 2,4-D. Both cultures were then incubated in darkness at 24 ± 1 °C for seven weeks and then exposed to light (16 h photoperiod at a light intensity of 70 μE $m^{-2}s^{-1}$) at 24 ± 1 °C for five weeks. When cream-colored calli appear, they are transferred to PGRs-free medium. Cultures are maintained under light (16 h photoperiod at a light intensity of 70 μE $m^{-2}s^{-1}$) at 24 ±1 °C for six weeks.

6. Procedure SM6: In vitro Organogenesis of Roots from Peach Leaf Explants.

Leaves from greenhouse-grown seedlings and mature, greenhouse-grown plants are disinfested by immersion for 30 seconds in 70% ethanol and then soaked for 10 min in a solution of 0.8% sodium hypochlorite with 2 drops per liter of Tween 20. They are then rinsed 3 times in sterile distilled water. Leaves are cut into transverse strips with a scalpel and explanted abaxial surface down onto treatment media. Each leaf produced 5-6 segments with the segment(s) from mid-leaf ranging in size from approximately 6 × 8 mm to 9 × 15 mm. Several punctures are made through each leaf piece. The growth regulator treatments added to MS medium [7] included combinations of kinetin at 0, 0.4, 1.2, 3.6, and 10.8 μM; with IBA or NAA at 0, 3.0, 6.0, 9.0 and 12.0 μM. PGRs were added to the basal medium prior to autoclaving. Root development was evaluated after 5 weeks of culture in the dark or light (45-50 μE $m^{-2}s^{-1}$ with a 16 h photoperiod) at 24 °C.

7. Procedure SM7: Improved Peach in vitro Shoot Proliferation.

Greenhouse-grown plants/cuttings are preferred as explant source for culture initiation, due to less incidences of microbial contamination and immediate robust regrowth, once shoot tips were placed on shoot proliferation medium. Shoots are cultured in LP basal medium [9] lacking myo-inositol and supplemented with vitamins 1.0 mg/L thiamine-HCL, 1.0 mg/L nicotinic acid, 1.0 mg/L pyridoxine-HCL, 4.0 mg/L glycine, 0.2 mg/L biotin and 2.0 mg/L Ca-pantothenate, phytohormones 4.5 μM BAP and 0.5 μM IBA, 3% sucrose, 0.6% Sigma agar (Sigma-Aldrich, St. Louis, Missouri, USA, A7921) and pH value adjusted to 5.8. Medium is sterilized in an autoclave at 121 °C for 20 min. Cultures are maintained at 25 °C under a 16 h photoperiod (50 μE $m^{-2}s^{-1}$) and subcultured to fresh medium every three weeks.

A two-step procedure is employed to improve in vitro shoots rooting and establishment in the greenhouse. In vitro shoots, of more than 2 cm length, are first transferred to LP medium (lacking myo-inositol) supplemented with 5.0 μM IBA and cultured for 10 days to induce root primordia. After that, to encourage root and shoot development, 5–7 shoots are moved to each Magenta GA7 vessels containing PGRs-free LP medium with a culture tube closure containing the fungus *Cladosporium sphaerospermum* strain TC09 (Figure 7f, g). Cultures are maintained at 25 °C under a 16 h photoperiod (50 μE $m^{-2}s^{-1}$) for 10 days. Afterwards, robust plantlets with well-developed root system are transplanted to 1020 plastic trays (11" W × 21.37" L × 2.44" D) containing potting soil mix (Metro Mix, 852-F52, Sun Gro® Horticulture, Seba Beach, Canada) (Figure 7h). Trays are covered

with translucent plastic covers and kept under a shaded area for two weeks. Acclimatized plants are then transplanted to 6-inch pots containing similar soil mix for further growth and development in the greenhouse (Figure 7i).

Details of fungal culture initiation and preparation are described previously [15]. Briefly, aqueous conidial suspension is prepared by first culturing the fungal conidia on MS plate for 1–2 weeks followed by collecting conidia in sterile 0.01% Triton X-100/water solution and adjusting density to 105 conidia per ml prior to use as inoculum. Aliquots of 5 ml warm, growth regulator-free MS medium are poured into open-end culture tube closures (Sigma C5791). Once solidified, 10 µl of TC09 conidial suspension is added onto the surface of the medium. One inoculated closure is placed in each Magenta GA7 vessel.

8. Procedure SM8: Bud Regeneration from in vitro Peach Nodal Explants.

Three to four weeks-old in vitro shoots are used. Elongated, healthy, thick and green (non-lignified) shoots are chosen, and all buds are removed with a scalpel. Axillary buds are removed by running the scalpel parallel to the stem. Shoots are cut into segments containing, at least one node and placed onto regeneration medium, consisting of QL macro and micronutrients, vitamins and organic compounds [2], 3% sucrose, 0.7% Purified agar, 6.7 µM BAP and 0.05 µM IBA. The pH is adjusted to 5.7 before autoclaving at 121 °C for 20 min.

References:

1. Gentile, A.; Monticelli, S.; Damiano, C. Adventitious shoot regeneration in peach (*Prunus persica* L., Batsch). *Plant Cell Rep.* **2002**, *20*, 1011–1016. DOI: 10.1007/s00299-002-0451-2
2. Quoirin, M.; Lepoivre, P. Improved media for *in vitro* culture of *Prunus* sp. *Acta Hortic.* **1977**, 437–442. DOI: 10.17660/actahortic.1977.78.54
3. Pérez-Tornero, O.; Burgos, L.; Egea, J. Introduction and establishment of apricot *in vitro* through regeneration of shoots from meristem tips. *Vitr. Cell. Dev. Biol. - Plant* **1999**, *35*. DOI: 10.1007/s11627-999-0087-9
4. Pérez-Tornero, O.; Burgos, L. *Apricot micropropagation*; Jain, S.M., Häggman, H., Eds.; 1st ed.; Springer: Dordrecht, The Netherlands. **2007**, pp. 267–278. DOI: 10.1007/978-1-4020-6352-7_25
5. Driver, J.A.; Kuniyuki, A.H. *In vitro* propagation of Paradox walnut rootstock. *HortScience* **1984**, *19*, 507–509.
6. Petri, C.; Scorza, R.; Srinivasan, C. Highly efficient transformation protocol for plum (*Prunus domestica* L.). *Methods Mol. Biol.* **2012**, *847*, 191–199. DOI: 10.1007/978-1-61779-558-9_16
7. Murashige, T.; Skoog, F. A revised medium for rapid growth and bioassays with tobacco tissue cultures. *Physiol. Plant.* **1962**, *15*, 473–497. DOI: 10.1111/j.1399-3054.1962.tb08052.x
8. Chée, R.; Pool, R.M. The effects of growth substances and photoperiod on the development of shoot apices of *Vitis* cultured *in vitro*. *Sci. Hortic. (Amsterdam).* **1982**, *16*, 17–27. DOI: 10.1016/0304-4238(82)90020-6
9. Quoirin, M.; Lepoivre, P.; Boxus, P. Un premier bilan de 10 années de recherches sur les cultures de méristèmes et la multiplication *in vitro* de frutiers ligneux. In *Compte rendu des recherches 1976-1977 EJ Rapports de synthèse. Station de cultures frutières et maraichères*; Gembloux, Belgium. **1977**, pp. 93–117.
10. Franks, T.; He, D.G.; Thomas, M. Regeneration of transgenic *Vitis vinifera* L. Sultana plants: genotypic and phenotypic analysis. *Mol. Breed.* **1998**, *4*, 321–333. DOI: 10.1023/A:1009673619456
11. Dhekney, S.A.; Li, Z.T.; Grant, T.N.L.; Gray, D.J. Somatic embryogenesis and genetic modification of *vitis*. In *Methods in Molecular Biology*; Humana Press Inc., 2016; Vol. 1359, pp. 263–277. DOI: 10.1007/978-1-4939-3061-6_11
12. Nitsch, J.P.; Nitsch, C. Haploid plants from pollen grains. *Science.* **1969**, *165*, 85–87. DOI: 10.1126/science.163.3862.85
13. Gamborg, O.L.; Miller, R.A.; Ojima, K. Nutrient requirements of suspension cultures of soybean root cells. *Exp. Cell Res.* **1968**, *50*, 151–158. DOI: 10.1016/0014-4827(68)90403-5
14. Dhekney, S.A.; Li, Z.T.; Compton, M.E.; Gray, D.J. Optimizing initiation and maintenance of *Vitis* embryogenic cultures. *HortScience* **2009**, *44*, 1400–1406. DOI: 10.21273/HORTSCI.44.5.1400

15. Li, Z.T.; Janisiewicz, W.J.; Liu, Z.; Callahan, A.M.; Evans, B.E.; Jurick, W.M.; Dardick, C. Exposure *in vitro* to an environmentally isolated strain TC09 of *Cladosporium sphaerospermum* triggers plant growth promotion, early flowering, and fruit yield increase. *Front. Plant Sci.* **2019**, *9*, 1959. DOI: 10.3389/fpls.2018.01959
